# Synthesis and Physicochemical Characterization of the Process-Related Impurities of Eplerenone, an Antihypertensive Drug

**DOI:** 10.3390/molecules22081354

**Published:** 2017-08-15

**Authors:** Iwona Dams, Agata Białońska, Piotr Cmoch, Małgorzata Krupa, Anita Pietraszek, Anna Ostaszewska, Michał Chodyński

**Affiliations:** 1 R&D Chemistry Department, Pharmaceutical Research Institute, Rydygiera 8, 01-793 Warsaw, Poland; p.cmoch@ifarm.eu (P.C.); m.krupa@ifarm.eu (M.K.); a.pietraszek@ifarm.eu (A.P.); a.ostaszewska@ifarm.eu (A.O.); m.chodynski@ifarm.eu (M.C.); 2 Faculty of Chemistry, University of Wrocław, Joliot-Curie 14, 50-383 Wrocław, Poland; agata.bialonska@chem.uni.wroc.pl

**Keywords:** selective aldosterone blocker, hypertension, steroids, eplerenone, impurities, spectroscopic methods, crystal structure

## Abstract

Two unknown impurities were observed during the process development for multigram-scale synthesis of eplerenone (Inspra^®^). The new process-related impurities were identified and fully characterized as the corresponding (7β,11α,17α)-11-hydroxy- and (7α,11β,17α)-9,11-dichloroeplerenone derivatives **12a** and **13**. Seven other known but poorly described in the literature eplerenone impurities, including four impurities A, B, C and E listed in the European Pharmacopoeia 8.4 were also detected, identified and fully characterized. All these contaminants result from side reactions taking place on the steroid ring C of the starting 11α-hydroxy-7α-(methoxycarbonyl)-3-oxo-17α-pregn-4-ene-21,17-carbolactone (**12**) and the key intermediate (7α,17α)-9(11)-enester **7**, including epimerization of the C-7 asymmetric center, oxidation, dehydration, chlorination and lactonization. The impurities were isolated and/or synthesized and fully characterized by infrared spectroscopy (IR), nuclear magnetic resonance spectroscopy (NMR) and high-resolution mass spectrometry/electrospray ionization (HRMS/ESI). Their ^1^H- and ^13^C-NMR signals were fully assigned. The molecular structures of the eight impurities, including the new (7β,11α,17α)-11-hydroxy- and (7α,11β,17α)-9,11-dichloroeplerenone related substances **12a** and **13**, were solved and refined using single-crystal X-ray diffraction (SCXRD). The full identification and characterization of these impurities should be useful for the quality control and the validation of the analytical methods in the manufacture of eplerenone.

## 1. Introduction

**Figure 1:**
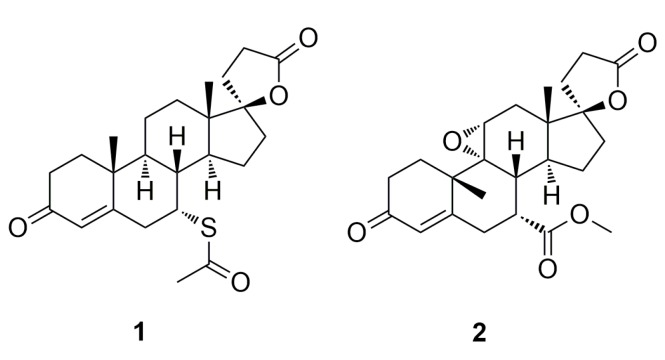
Chemical structures of the aldosterone receptor antagonists spironolactone (Aldactone^®^, **1**) and eplerenone (Inspra^®^, **2**).

Eplerenone (**2**, Figure 1) is a cardiovascular drug indicated for the treatment of essential hypertension and congestive heart failure that, in contrast to its predecessor spironolactone **1** (Figure 1) demonstrates a high degree of selectivity for the aldosterone receptor and a low-binding affinity for progesterone and androgen receptors [1,2,3,4,5,6,7,8]. As a result of the presence of a 9,11-epoxide group in the eplerenone structure, its selectivity for the aldosterone receptor is enhanced and the drug minimizes the risk of adverse hormonal effects and provides important clinical benefits not previously available with spironolactone **1**. Treatment with eplerenone is associated with reductions in blood pressure and improved survival (15% reduction in total mortality) for patients with heart failure who are in stable condition after a myocardial infarction. The product was originally developed by scientists at Ciba-Geigy AG (Basel, Switzerland) and launched in the US in 2003 by Pharmacia (Sandwich, Kent, UK) under the trade name Inspra^®^.

Eplerenone contains sensitive 9,11α-epoxide, 17α-γ-lactone and 7α-carbomethoxy moieties that, depending on the conditions, may hydrolyze or epimerize to afford degradation or epimerization products [9,10,11,12,13]. The SCXRD structure of eplerenone confirms the relative *cis* configuration of the 9α,11α-epoxide ring and the 7α-carbomethoxy substituent [14,15,16,17]. Manufacture of eplerenone is always accompanied by side reactions leading to the unwanted impurities that vary with the different starting materials and reaction conditions used. Most of the patents and literature information dealing with the preparation of eplerenone are based on the use of canrenone derivatives. As stereogenic centers in eplerenone precursors give rise to various process-related impurities, including diastereo- and regioisomers of starting materials, intermediates, by-products and the final drug substance, the manufacture of eplerenone with the required stereochemistry and pharmaceutical grade purity is a significant challenge. In designing a synthesis of eplerenone from canrenone derivatives, the principal challenges are the stereoselective introduction of the carbomethoxy substituent at the C-7α position of the steroid skeleton and the regioselective dehydration of the 11α-hydroxy group [1,9,16,17,18,19,20,21].

**Scheme 1:**
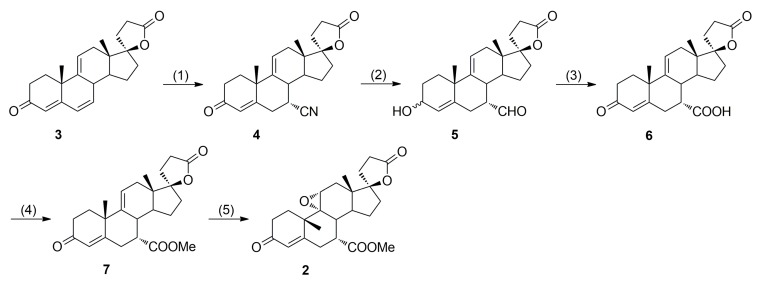
The original eplerenone synthesis from Ciba-Geigy AG (Basel, Switzerland) [17,18]. *Reagents and Conditions*: (1) Et_2_AlCN, THF; (2) DIBAL-H, benzene; (3) CrO_3_, H_2_SO_4_, acetone; (4) CH_2_N_2_, CH_2_Cl_2_; (5) m-CPBA, CH_2_Cl_2_ or H_2_O_2_, Cl_3_CCN, K_2_HPO_4_, CH_2_Cl_2_.

In 1984, Grob et al. [17,18] from Ciba-Geigy AG accomplished the first synthesis of eplerenone by employing Nagata hydrocyanation of Δ^9(11)^-canrenone (**3**) as the key step (Scheme 1), but with moderate stereoselectivity in the 7α-cyano derivative **4** formation (7α/7β ≈ 4:1) necessitating tedious column chromatographic separations. The original EP 122232 B1 patent does not disclose any information with respect to the purity of the material obtained, its purification to pharmaceutical-grade or the removal of impurities.

**Scheme 2:**
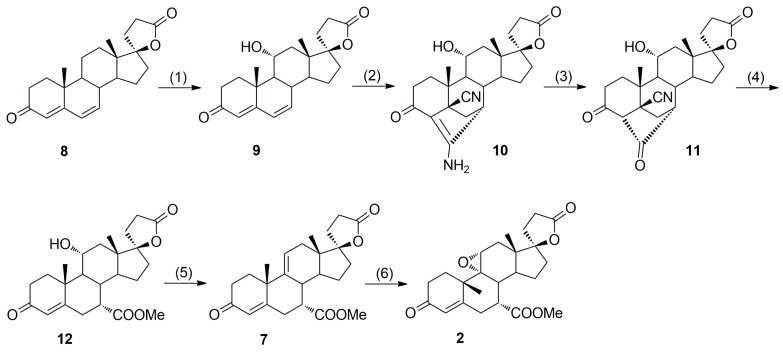
Synthesis of EP from G. D. Searle & Co. (Chicago, IL, USA) [9]. *Reagents and Conditions*: (1) *Aspergillus ochraceus*; (2) LiCl, DMF, Et_3_N, Me_2_CO, (CH_3_)_2_C(OH)CN; (3) HCl, MeOH, H_2_O; (4) MeONa, MeOH; (5) SO_2_Cl_2_, ImH, THF; (6) H_2_O_2_, K_2_HPO_4_, Cl_3_CCONH_2_.

In alternative eplerenone synthesis by Ng et al. [9], the 7α-carbomethoxy group was introduced stereoselectively via 4,6-bishydrocyanation of 11α-hydroxycanrenone (**9**, Scheme 2), but regioselective dehydration of the intermediate (7α,11α,17α)-11-hydroxyester **12** was problematic giving the regioisomeric (7α,17α)-11(12)-enester **7b** apart from the main (7α,17α)-9(11)-enester **7** product (Table 1). The 7α/7β diastereoisomeric purity of (7α,11α,17α)-**12** obtained by the cleavage of diketone **11** with sodium methoxide in refluxing methanol was not reported; however, the literature sources on syntheses of related 7α-carboalkoxy stereoidal spirolactones provide information on their 7β-carboalkoxy diastereoisomers resulting from basic epimerization of the steroid C-7α asymmetric center under the same conditions as used for diketone **11** cleavage [22].

When the authors repeated the preparation taught in WO 9825948 A2 it was observed that the purity of the resulting eplerenone was low and attempts to purify the resulting product to a level meeting the requisite specifications for use as a pharmaceutical active were unsuccessful. Although the epimeric 11-hydroxyester (7β,11α,17α)-**12a** was not reported by Ng et al. [9], or any other literature sources, it contaminated the main (7α,17α)-9(11)-enester **7** product. In particular, the regioisomeric (7α,11α,12α,17α)-11,12-epoxyester **2b** (Imp. E), the new (7α,11β,17α)-9,11-dichloro derivative **13** and the 7α,9:21,17-dicarbolactone **14** (Imp. A) were difficult to remove from the final product. All these contaminants resulted from side reactions taking place on the steroid ring C of the (7α,11α,17α)-11-hydroxy derivative (**12**) and the key intermediate (7α,17α)-9(11)-enester **7**, including epimerization of the C-7α asymmetric center, oxidation, dehydration, chlorination and lactonization.

Recently, we have developed an improved, scalable, cost-effective and environment-friendly technology for the industrial-scale synthesis of eplerenone (**2**) from commercially available 11α-hydroxy-7α-(methoxycarbonyl)-3-oxo-17α-pregn-4-ene-21,17-carbolactone (**12**), based on the last two steps of the general route described by Ng et al. (Scheme 2) [9]. During the process development, two new and seven known process-related impurities of eplerenone were observed, and/or synthesized and fully characterized, including four impurities A, B, C and E listed in the European Pharmacopoeia 8.4 [23]. The new impurities were identified as 11α-hydroxy-7β-(methoxycarbonyl)-3-oxo-17α-pregn-4-ene-21,17-carbolactone (**12a**) and 9,11β-dichloro-7α-(methoxycarbonyl)-3-oxo-17α-pregn-4-ene-21,17-carbolactone (**13**). To the best of our knowledge, the 11-hydroxyester **12a** and the 9,11-dichloro derivative **13** are new compounds that have never been identified before.

The syntheses of the starting (7α,11α,17α)-11-hydroxyester **12** and the key intermediate (7α,17α)-9(11)-enester **7** are broadly described in the literature sources [9,16,17,18,19,20,21,24]; however, their comprehensive structure elucidation and confirmation is still missing and SCXRD studies are reported here for the first time. As starting materials and intermediates in active pharmaceutical ingredient (API) synthesis often afford numerous impurities affecting the quality of the final drug product, their comprehensive structural elucidation and confirmation is essential for impurities identification and characterization. A complete physicochemical characterization, not only for an API, but also starting materials and key synthetic intermediates, became a requirement of both the U.S. Food and Drug Administration (FDA) and the European Medicine Agency (EMA). The five other impurities listed in Table 1, i.e., the isomeric (7β,17α)-9(11)-enester **7a** and (7α,17α)-11(12)-enester **7b**, the isomeric (7β,11α,17α)-9,11-epoxyester **2a** (Imp. E) and (7α,11α,12α,17α)-11,12-epoxyester **2b** (Imp. B) and the 7α,9:21,17-dicarbolactone **14** (Imp. A), were mentioned elsewhere in the literature, mainly as part of HPLC studies; however, their syntheses, methods of removal from the final product and comprehensive structural elucidation and confirmation were not disclosed [9,12]. The determination of a drug substance impurity profile, including starting materials, by-products, intermediates and potential degradation products, is critical for the safety assessment of API and manufacturing process thereof. In any API, it is necessary to study the impurity profile, and control it during the preparation of the pharmaceutical. As indicated in the ICH guidelines, any impurity, formed at a level of ≥0.10% with respect to the API, should be identified, synthesized and characterized thoroughly [25,26]. Only two eplerenone impurities, i.e., 7α,9:21,17-dicarbolactone **14** (Imp. A) and 11,12-epoxy ester **2b** (Imp. B), are accepted at a level greater than 0.10%, i.e., maximum 0.3%, in accordance with pharmacopoeial acceptance criteria [22].

The present paper deals with synthesis, physicochemical characterization and comprehensive structural elucidation and confirmation of the process-related eplerenone impurities found in samples of the key intermediate (7α,17α)-9(11)-enester **7** and eplerenone bulk drug substance manufactured according to the modified and optimized procedure described in Searle Co. patent [9] (Scheme 2, **12** as starting material). Apart from the starting material **12**, the key intermediate enester **7** and the final product **2**, seven process-related eplerenone impurities **2a**–**b**, **7a**–**b**, **12a**, **13** and **14** were fully characterized by IR, NMR and MS. The molecular structures of the epimeric 11-hydroxyesters (7α,11α,17α)-**12** and (7β,11α,17α)-**12a**, the isomeric (7α,17α)-9(11)-enester **7**, (7β,17α)-9(11)-enester **7a** and (7α,17α)-11(12)-enester **7b**, the isomeric (7β,11α,17α)-9,11-epoxyester **2a** and (7α,11α,12α,17α)-11,12-epoxyester **2b**, and the new (7α,11β,17α)-9,11-dichloro derivative **13**, were solved and refined using SCXRD. The full identification and characterization of these compounds should be useful for the quality control and the validation of the analytical methods in the manufacture of eplerenone.

## 2. Results and Discussion

Dehydration of 11-hydroxy steroids is commonly used for the introduction of the 9,11-double bond into the steroid skeleton. In a preferred technological embodiment, the (7α,17α)-9(11)-enester **7** is synthesized directly by in situ replacement of the 11α-hydroxy group of the ester (7α,11α,17α)-**12** with halogen followed by thermal 9,11-dehydrohalogenation. The nucleophilic substitution of the 11α-hydroxy group is effected by reaction with sulfuryl halide at about −70 °C, after which a hydrogen halide scavenger is added. In manufacturing technology of eplerenone developed by the authors, the 11α-hydroxyester **12** and imidazole were dissolved simultaneously in anhydrous tetrahydrofuran and cooled to −10 °C. Sulfuryl chloride was added slowly and the reaction mixture was allowed to warm to room temperature, and then stirred for a time sufficient to complete the elimination reaction, typically about 1 h. The key intermediate **7** was isolated in a crude form by dichloromethane extraction followed by evaporation of the solvent and recrystallized twice, from ethanol and a mixture of dichloromethane/diethyl ether respectively, to give the pure (7α,17α)-9(11)-enester **7** (71% yield) as white crystals.

The dehydration step produced the three main side products, i.e., the regioisomeric (7β,17α)-11(12)-enester **7b**, the new (7α,11β,17α)-9,11-dichloro derivative **13** and the 7α,9:21,17-dicarbolactone **14** (Imp. A), which were isolated chromatographically from the recrystallization mother liquors of **7** by elution with varying mixtures of ethyl acetate/dichloromethane. Further gradient elution with acetonitrile/toluene afforded the new 11-hydroxyester (7β,11α,17α)-**12a**, which was isolated in only a small yield, apart from the unreacted (7α,11α,17α)-**12** isomer used as the starting material.

The commercial 11-hydroxyester (7α,11α,17α)-**12** was synthesized according to the procedure described by Ng et al. [9] and the chemical purity declared by the manufacturer was 98%. The novel 11-hydroxyester (7β,11α,17α)-**12a** was not previously detected by chromatographic or spectroscopic methods in its commercially available (7α,11α,17α)-**12** isomer. However, the literature data on syntheses of related 7α-carboalkoxy steroidal spirolactones [22] allowed us to assume that the new 11-hydroxyester (7β,11α,17α)-**12a** originates from the fourth step of the Scheme 2 synthesis in which sodium methoxide reacts with diketone **11** to afford the (7α,11α,17α)-**12** isomer as the main product of the cleavage, which is contaminated with the (7β,11α,17α)-**12a** epimer resulting from the competitive basic epimerization of **12**. The 11-hydroxyester (7β,11α,17α)-**12a** was synthesized independently by basic epimerization of the (7α,11α,17α)-**12** isomer. As expected, sodium methoxide in methanol solution converted the C-7α ester **12** into an epimeric mixture of the C-7α **12** and C-7β **12a** esters. The pure C-7β ester **12a** was isolated chromatographically from the crude mixture of epimers **12** and **12a** by 10–50% acetonitrile/toluene gradient elution to give (7β,11α,17α)-**12a** (49% yield) as a white solid. A sample of **12a** was also isolated by column chromatography from recrystallization mother liquors of the commercial 11-hydroxyester (7α,11α,17α)-**12**. Even a small amount of the newly detected (7β,11α,17α)-11-hydroxyester **12a** is hardly separable from its (7α,11α,17α)-**12** isomer by large scale chromatography or recrystallization, therefore, the commercial 11-hydroxyester (7α,11α,17α)-**12** was used further without purification. As the two 11-hydroxyesters of the C-7α and C-7β series, respectively **12** and **12a**, are crucial for impurities formation during multi-gram scale synthesis of eplerenone, they were subjected to comprehensive structural elucidation and confirmation.

**Figure 2:**
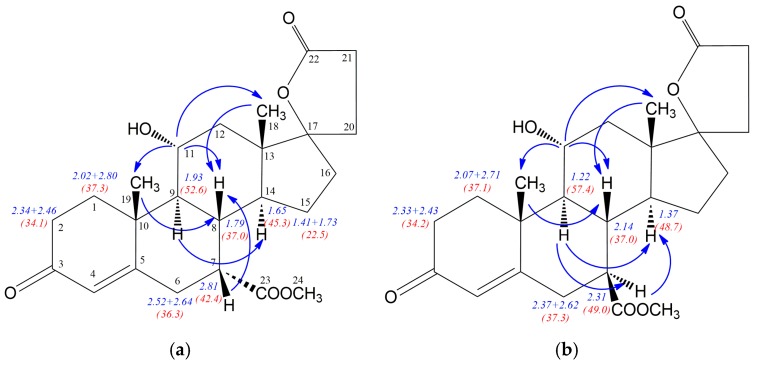
Crucial ^1^H- and ^13^C-NMR chemical shifts and NOE effects for 11-hydroxyesters: (**a**) (7α,11α,17α)-**12**; (**b**) (7β,11α,17α)-**12a**.

The [M + Na]^+^ values, *m*/*z* 439.2110 and 439.2080, obtained for both 11-hydroxyesters **12** and **12a** correspond to C_24_H_32_O_6_Na. The NMR data for epimeric hydroxyesters **12** and **12a** are given in Table 2. The ^1^H-/^13^C-NMR chemical shifts assignment was made after careful and precise 2D NMR spectra analysis and the data obtained for the known isomer **12** are in full compliance with those presented by Preisig et al. [24]. The 2D NOESY experiments allowed discrimination between epimeric structures **12** and **12a** and fully confirmed the stereochemistry at the C-7 atom in both isomers. Blue arrows in Figure 2 show the most important NOE effects involving H7 proton, simultaneously clearly indicating the 7α or 7β positioned carbomethoxy group. The strong H7-H8 NOE effect is observed for the compound **12** with β positioned H7 proton, whereas α situated H7 proton in **12a** is involved in two significant H9-H7 and H7-H14 NOE effects. Additionally, the strong NOE effect between H9 and H14 protons for both **12** and **12a** epimers is observed. Comparison of the NMR data for epimeric structures **12** and **12a** revealed that some of the ^1^H/^13^C nuclei shieldings within the steroid rings B, C and D are related to the α or β configuration of the C-7 atom. The ^1^H shielding increase of 0.5 ppm for H7, 0.71 ppm for H9 and 0.28 ppm for H14 nuclei is observed when passing from the compound **12** with 7α positioned carbomethoxy group to its 7β epimer **12a**. Additionally, the change of configuration at the C7 is accompanied by H8 (0.35 ppm) shielding decrease. Simultaneously, both diasterotopic H15 protons of the epimer **12a** with 7β positioned carbomethoxy group became equal having the same proton chemical shift (1.49 ppm). In the case of ^13^C-NMR data the opposite effect is observed, the transition from **12** to **12a** leads to the shielding decrease of 6.6 ppm for C7, 4.8 ppm for C9 and 3.4 ppm for C14 nuclei. Surprisingly, the change of configuration from 7α in **12** to 7β in **12a** is related with no change of the shielding of the C8 nucleus and decreasing of shielding by 2.7 ppm for C23 carbon of the carbomethoxy substituent.

**Figure 3:**
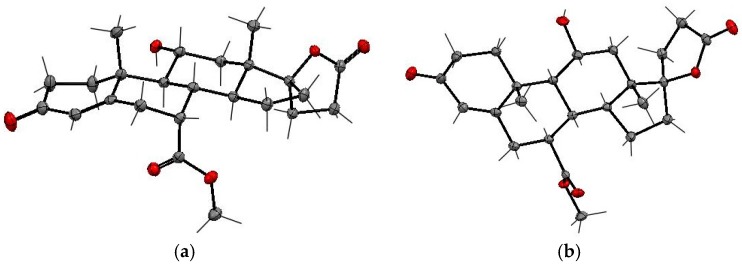
Molecular structure of the 11-hydroxyesters: (**a**) (7α,11α,17α)-**12** and (**b**) (7β,11α,17α)-**12a**.

The NMR structure assignments of the isomeric 11-hydroxyesters (7α,11α,17α)-**12** and (7β,11α,17α)-**12a** were confirmed by X-ray analysis (Figure 3 and Table 3). The SCXRD structures confirmed the relative *cis*- and *trans*-configurations of the 11α-hydroxy and 7α-carbomethoxy substituents in **12** and **12a**, respectively. The carbomethoxy group is situated at the C-7α position in **12** and the C-7β position in **12a**, whereas the hydroxy group adopts the C-11α position in both **12** and **12a** isomers. The dehydration of 11α-hydroxy steroids leads predominantly to the formation of the double bond between C-9 and C-11 carbons of the steroid skeleton. As expected, the (7α,17α)-9(11)-enester **7** was the main product of the 11-hydroxyester **12** dehydration with sulfuryl chloride in the presence of imidazole; however, a considerable amount of the regioiosmeric (7α,17α)-11(12)-enester **7b**, as a result of competitive 11,12-dehydration of **12**, was also isolated from the recrystallization mother liquors of **7**.

Additionally, the 9,11-dehydration of the residual 11-hydroxyester (7β,11α,17α)-**12a** contaminating the starting **12** isomer afforded the epimeric (7β,17α)-9(11)-enester **7a**, which was also isolated chromatographically from recrystallization mother liquors of **7**. It was synthesized independently by epimerization of the (7α,17α)-9(11)-enester **7** with sodium methoxide in methanol, or dehydration of the 11-hydroxyester (7β,11α,17α)-**12a** with sulfuryl chloride and imidazole, and then purified by column chromatography with 5–20% ethyl acetate/dichloromethane gradient elution to give the pure (7β,17α)-9(11)-enester **7a**, with 37% or 85% yield respectively, as a white solid.

**Figure 4:**
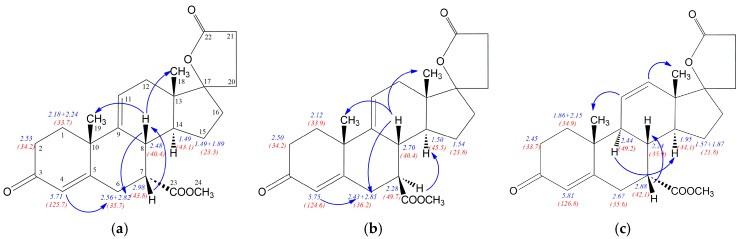
Crucial ^1^H- and ^13^C-NMR chemical shifts and NOE effects for esters: (**a**) (7α,17α)-9(11)-ene **7**; (**b**) (7β,17α)-9(11)-ene **7a**; (**c**) (7α,17α)-11-ene **7b**.

The [M + Na]^+^ values, *m*/*z* 421.1986, 421.2006, and 421.1988, obtained for the free isomeric enesters **7**, **7a** and **7b** correspond to C_24_H_30_O_5_Na. The full NMR data confirming the structure of the known enester **7** were reported by Grob et al. [17]; however several ^1^H and ^13^C signals were assigned reversibly. The proper ^1^H/^13^C chemical shift assignment for isomeric enesters **7**, **7a** and **7b** was based on the careful analysis of 2D NMR experiments and is given in Table 4. Similarly to the epimeric hydroxyesters **12** and **12a**, the NOE effects involving H7 proton allowed distinction between 7α (**7** and **7b**) and 7β (**7a**) diastereoisomers (Figure 4). The strong H7-H8 NOE effect was noted for isomers **7** and **7b** with β positioned H7 proton, whereas α situated H7 in **7a** is involved in H7-H14 NOE effect disturbed by H7-H15 interaction. Additionally, the significant H9-H14 NOE effect for **7b** regioisomer was observed, while H8 proton of the epimers **7** and **7b** is involved in H8-H6 interaction. Similarly to the epimeric hydroxyesters **12** and **12a**, some of the ^1^H/^13^C nuclei shieldings within the steroid rings B, C and D are related to the α or β configuration of the C-7 atom. The ^1^H shielding increase of 0.7 ppm for H7 and 0.22 ppm for H8 nuclei was observed when passing from the compound **7** with 7α positioned carbomethoxy group to its 7β epimer **7a**. Similarly to **12a**, both diasterotopic H15 protons of the epimer **7a** with 7β positioned carbomethoxy group became equal having the same proton chemical shift (1.54 ppm). In the case of ^13^C-NMR the opposite effect is observed, the transition from **7** to **7a** leads to the shielding decrease of 5.9 ppm for C7 and 2.4 ppm for C14 nuclei. The change of configuration from 7α in **7** to 7β in **7a** is related with no change of the shielding of the C8 nucleus and decreasing of shielding by 2.3 ppm for C23 carbon of carbomethoxy substituent. The dehydration of the epimeric 11-hydroxyesters **12**/**12a** to the corresponding 9(11)-enesters **7**/**7a** caused the strong ^1^H shielding decrease of 89.7 ppm/84.7 ppm for C9 and 49.8 ppm/51.9 ppm for C11 carbons, respectively. The transition from **12**/**12a** to **7**/**7a** also resulted in medium shielding increase of 10.5/10.8 ppm for C12 and shielding decrease of 8.8/7.3 ppm for C19 and 3.4/3.4 ppm for C8, whereas for C10 (1.1/1.3 ppm), C13 (1.7/1.9 ppm) and C14 (2.2/3.2 ppm) a weak increasing effect was observed. The competitive dehydration of **12** to the regioisomeric 11,12-enester **7b** resulted in the strong ^1^H shielding decrease of 57.3 ppm for C11 and 90.4 ppm for C12 nuclei. Similarly to the hydroxyesters **12** and **12a**, the change of configuration from 7α in **7** to 7β in **7a** is related with no change of the shielding of the C8 nucleus and decreasing of shielding by 2.3 ppm for C23 carbon of the carbomethoxy substituent. Additionally, the shielding increase of 4.5 ppm for C8 carbon was observed when passing from epimeric 9(11)-enesters **7** and **7a** to regioisomeric 11-enester **7b**.

**Figure 5:**
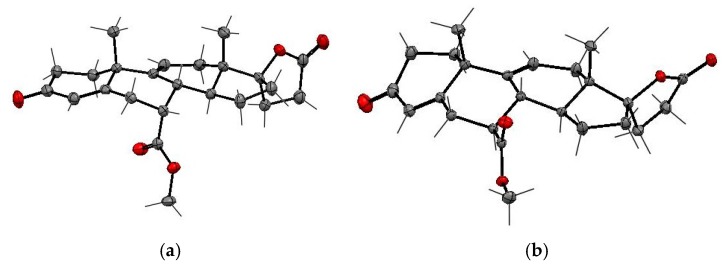
Molecular structure of the isomeric 9(11)-enesters: (**a**) (7α,17α)-**7** and (**b**) (7β,17α)-**7a**.

The NMR structure assignments of the isomeric (7α,17α)-9(11)-enester **7**, (7β,17α)-9(11)-enester **7a** and (7α,17α)-11(12)-enester **7b** were confirmed by X-ray analysis (Figure 5 and Table 5 and Table 6). The SCXRD structures confirmed the presence of the 9,11-double bond in **7** and **7a** and the 11,12-double bond in **7b**. The carbomethoxy group is situated at the C-7α position in **7** and **7b** and at the C-7β position in **7a**.

Sulfuryl chloride used for dehydration of 11α-hydroxy steroids is also known as a chlorinating agent and, under some conditions, applied for chlorination of steroid double bonds [27,28]. Indeed, the novel (7α,11β,17α)-9,11-dichloro derivative **13**, formed as a result of competitive chlorine addition to the newly formed 9,11-double bond of the key intermediate (7α,17α)-9(11)-enester **7**, was isolated chromatographically from the recrystallization mother liquors of **7**. It was also synthesized by chlorination of the pure enester **7** with sulfuryl chloride in the presence of pyridine in chlorobenzene, and then purified by column chromatography with 5–30% EtOAc/CH_2_Cl_2_ gradient elution to give the pure (7α,11β,17α)-9,11-dichloro derivative **13** (41% yield) as a white solid. The novel 9,11-dichloro impurity **13** was formed by the nucleophilic attack of a chloride anion on the intermediate chloronium cation, a structural analogue of the eplerenone epoxide ring. No other isomers of **13** were obtained.

The [M + Na]^+^ value, *m*/*z* 491.1386 obtained for the 9,11-dichloroderivative **13** corresponds to C_24_H_30_O_5_NaCl_2_. The stereochemistry at the C9 and C11 carbons of the dichloro derivative **13** was established on the basis of 1D/2D NOESY experiments, which indicated the relative *trans* configuration of the C-9α and C-11β positioned chlorine atoms. The addition of chlorine to the 9,11-double bond of enester **7** caused significant changes in the ^1^H/^13^C nuclei shieldings within the steroid rings A, B, C and D of the dichloro derivative **13** (Table 7).

**Figure 6:**
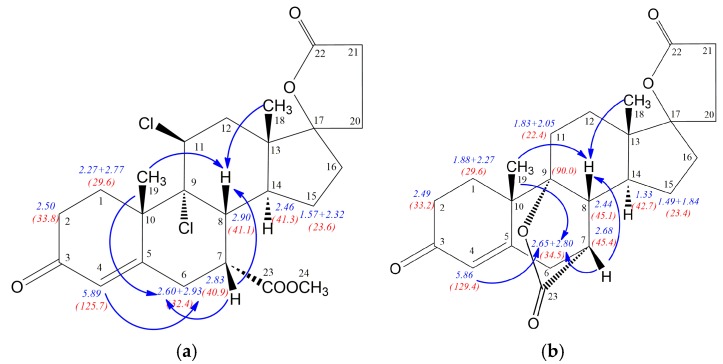
Crucial ^1^H- and ^13^C-NMR chemical shifts and NOE effects for: (**a**) (7α,11β,17α)-9,11-dichloro derivative **13**; (**b**) 7α,9:21,17-dicarbolactone **14**.

**Figure 7:**
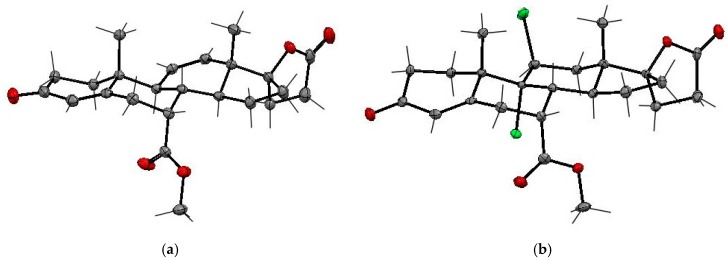
Molecular structure of (**a**) (7α,17α)-11-enester **7b** and (**b**) (7α,11β,17α)-9,11-dichloro derivative **13**.

The strong shielding increase of 55.7 ppm for C9 and 59.5 ppm for C11 nuclei was observed when passing from **7** to **13**. Minor shielding increase of 4.1 ppm for C1, 2.9 ppm for C5, 3.3 ppm for C6, 2.9 ppm for C7 and 1.8 ppm for C14 and shielding decrease of 6.6 ppm for C10 nuclei were also noted. The introduction of chlorine into C9 and C11 positions of **7** also resulted in shielding increase of 0.97 ppm for H11 and shielding decrease of 0.18 ppm for H4, 0.42 ppm for H8 and 0.97 for H14 nuclei in dichloro derivative **13**. Noteworthy, the diasterotopic effect observed for H15 protons of **7** (0.4 ppm) increased to 0.75 ppm for the same protons in **13**. Similarly to the other compounds with 7α-carbomethoxy group, the β positioned H7 proton in dichloro derivative **13** is involved in strong H7-H8 NOE effect (Figure 6). The NMR structure assignment of the novel (7α,11β,17α)-9,11-dichloro derivative **13** was confirmed by X-ray analysis (Figure 7 and Table 5). The SCXRD structure confirmed the presence of the two chlorine atoms at the C-9α and C-11β positions of the steroid ring C in the relative *trans* configuration and the C-7α positioned carbomethoxy substituent.

The acidic conditions applied for the dehydration of the 11α-hydroxy group in **12** resulted in competitive lactonization between the hydroxyl and carbomethoxy groups and lead to 7α,9:21,17-dicarbolactone **14** (Imp. A), which tends to be formed even in the presence of traces of free water. The dicarbolactone **14** was isolated chromatographically from the recrystallization mother liquors of 7. It was also synthesized independently from the mesylate of the starting 11α-hydroxyester **12** by reaction with acetic acid in the presence of sodium acetate, and then purified by column chromatography with 5–30% ethyl acetate/dichloromethane gradient elution to afford the pure 7α,9:21,17-dicarbolactone **14** (83% yield) as a white solid.

The [M + Na]^+^ value, *m*/*z* 407.1847, obtained for the dicarbolactone **14** corresponds to C_23_H_28_O_5_Na. The ^1^H-/^13^C-NMR chemical shifts assignment for dilactone **14** is presented in Table 7. The detailed analysis of 2D NMR spectra, especially ^1^H-^13^C HMBC and NOESY experiments, unambiguously confirmed the structure consisting of the two γ-lactone rings. The second γ-lactone moiety results from internal lactonization between 9α-hydroxy and 7α-carbomethoxy groups of intermediate ester, formed by water addition to the 9,11-double bond of enester **7** during the dehydration step of **12**. This significant change in the steroid structure entails numerous changes in the ^1^H/^13^C nuclei shielding. The shielding increase of 46.8 ppm for C11 and 16.5 ppm for C12 and decrease of 37.4 ppm for C9 nuclei were observed when passing from hydroxyester **12** to the dicarbolactone **14**. Minor shielding increase of 7.7 ppm for C1 and 6.2 ppm for C5 was also noted, whereas for C8 shielding decrease of 8.1 ppm was observed. Similarly to the other compounds of the series with 7α positioned carbomethoxy group, the H7 proton of the dicarbolactone **14** is involved in two strong H7-H8 and H6-H7 NOE effects (Figure 6).

The purification of the key intermediate (7α,17α)-9(11)-enester **7** by large-scale recrystallization only partially removes the impurities formed during the dehydration of the starting 11α-hydroxyester **12**. Thus, in a preferred technological embodiment, the key intermediate enester **7** was isolated in a crude form by dichloromethane extraction followed by evaporation of the solvent, and used directly in the following step of the process, which was the epoxidation of the 9,11-double bond to produce the eplerenone. Thus, the crude enester **7** was dissolved in dichloromethane and epoxidized with hydrogen peroxide in the presence of disodium hydrogen phosphate and trichloroacetamide. The crude product was recrystallized from ethanol and 2-butanone to give the pharmaceutical grade eplerenone. Apart from the (7α,11β,17α)-9,11-dichloro derivative **13** and the 7α,9:21,17-dicarbolactone **14** (Imp. A), which were present in the crude enester **7**, the epoxidation step produced the two further impurities, i.e., the isomeric (7β,11α,17α)-9,11-epoxyester **2a** and (7α,11α,12α,17α)-11,12-epoxyester **2b**, which were isolated chromatographically from the recrystallization mother liquors of **2**. The trichloroacetamide is known to preferentially epoxidize the more highly substituted double bond, e.g., the 9,11-olefin. In the case of the crude 9,11-enester **7** contaminated with the 11,12-enester **7b**, the 9,11-epoxide should be formed with the minimal competitive epoxidation of the **7b** isomer yielding the 11,12-epoxide as the by-product. As expected, the chromatography of the recrystallization mother liquors of **2** with 1–10% acetone/dichloromethane gradient elution gave the (7α,11α,12α,17α)-11,12-epoxyester **2b**, which was undoubtedly formed via epoxidation of the (7α,17α)-9(11)-enester **7b**. It was also synthesized independently by epoxidation of the enester **7b** with hydrogen peroxide in the presence of disodium hydrogen phosphate and trichloroacetamide, and purified by column chromatography to afford the pure 11,12-epoxyester **2b** (60% yield) as a white solid. Further gradient elution of the recrystallization mother liquors of **2** with 1–10% acetone/dichloromethane gave the isomeric (7β,11α,17α)-9,11-epoxyester **2a**, which was formed by epoxidation of the (7β,17α)-9(11)-enester 7a contaminating the crude intermediate enester **7**. It was also synthesized independently by epoxidation of the enester **7a** with hydrogen peroxide in the presence of disodium hydrogen phosphate and trichloroacetamide, or basic epimerization of eplerenone (**2**), and then purified by column chromatography to give the pure (7β,11α,17α)-9,11-epoxyester **2a**, with 69% or 49% yield respectively, as a white solid. The recrystallization of the crude eplerenone (**2**) from ethanol removes the new 9,11-dichloro derivative **13**, the isomeric (7β,11α,17α)-9,11-epoxyester **2a** and the residuals of the unreacted isomeric (7β,17α)-9(11)-enester **7a** and (7α,17α)-9(11)-enester **7b**. The recrystallization from 2-butanone removes the 7α,9:21,17-dicarbolactone **14** (Imp. A) and affords the pharmaceutical grade eplerenone (**2**).

**Figure 8:**
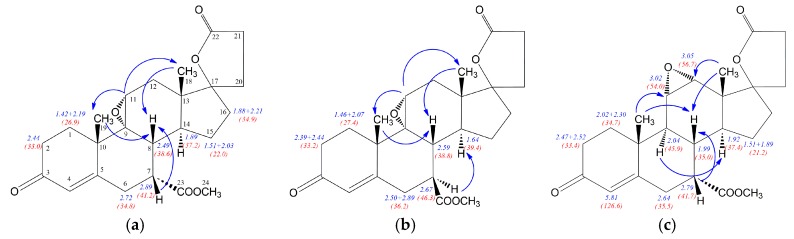
Crucial ^1^H- and ^13^C-NMR chemical shifts and NOE effects for: (**a**) (7α,11α,17α)-9,11-epoxyester **2**; (**b**) (7β,11α,17α)-9,11-epoxyester **2a** and (**c**) (7α,11α,12α,17α)-11,12-epoxyester **2b**.

The NMR data of eplerenone were presented in literature sources many times; however the full and correct ^1^H/^13^C chemical shifts assignment confirming its structure has never been done. Although the complete assignment of ^1^H-/^13^C-NMR signals was presented by Grob et al. [17], some of the signals were assigned reversibly. The careful analysis of 2D NMR experiments, including HSQC, HMBC and NOESY measurements, allowed to assign signals to the corresponding protons and carbons of the isomeric epoxy esters **2**, **2a** and **2b** unambiguously, and thus confirm the structure of eplerenone and its isomers (Table 8). Blue arrows in Figure 8 show the most important NOE effects involving H7 proton, simultaneously indicating the 7α or 7β positioned carbomethoxy group. The strong H7-H8 NOE effect is observed for the compounds **2** and **2b** with β positioned H7 proton, whereas α situated H7 proton in **2a** is involved in the strong interaction with H14. The epoxidation of the 9,11-double bond caused several shielding effects observed for ^1^H/^13^C nuclei within the steroid rings A, B and C. The strong shielding increase of 77.1/75 ppm for C9, 67.6/66.8 ppm for C11 carbons and 2.5/2.4 ppm for H11 protons was observed when passing from enesters **7**/**7a** to epoxides **2**/**2a**, respectively. Minor shielding increase of 7/6.5 ppm for C1, 5.9/6.1 ppm for C14, 2.6/3.4 for C7, 1.8/1.6 ppm for C8, 2/2 ppm for C12 and 0.7/0.4 for C13 carbons was also noted. The transition from enesters **7**/**7a** to epoxides **2**/**2a** caused shielding increase of 0.76/0.66 ppm for one of the H1 protons and 0.36/0.31 ppm for one of the H12 protons, whereas for H-14 protons shielding decrease of 0.4/0.14 ppm was noted. Similarly to the epimeric hydroxyesters **12**/**12a** and enesters **7**/**7a**, some of the ^1^H/^13^C nuclei shielding within the steroid rings B, C and D are related to the α or β configuration of the C7 atom. The ^1^H shielding increase of 0.22 ppm for H7 and 0.25 ppm for H14 nuclei is observed when passing from the compound **2** with 7α positioned carbomethoxy group to its 7β epimer **2a**. Additionally, the change of configuration at the C7 is accompanied by weak shielding decrease of 0.1 ppm for H8. Simultaneously, both diasterotopic H15 protons of the epimer **2a** with 7β positioned carbomethoxy group became equal having the same proton chemical shift (1.51 ppm). In the case of ^13^C-NMR data the opposite effect is observed, the transition from **2** to **2a** leads to the shielding decrease of 5.1 ppm for C7, 1.9 ppm for C9 and 2.2 ppm for C14 nuclei. The change of configuration from 7α in **2** to 7β in **2a** is also related with shielding decrease of 2.1 ppm for C23 carbon of the carbomethoxy substituent. The competitive epoxidation of the 11,12-enester **7b** to the 11,12-epoxyester **2b** resulted in the strong shielding increase of 76.1 ppm for C11 and 77.1 ppm for C12 nuclei, whereas for C9 (3.3 ppm), C18 (4.4 ppm) and C14 (6.7 ppm) medium effects were observed. The strong shielding increase of 2.57 and 2.84 ppm was noted for H11 and H12 protons, respectively. Minor changes in increasing of shielding for H7 (0.1 ppm), H8 (0.24 ppm) and H9 (0.4 ppm) protons were also observed. Similarly to the enesters **7**, **7a** and **7b**, the shielding increase of 3.6 ppm and 3.8 ppm for C8 carbon was noted when passing from epimeric 9,11-epoxides **2** and **2a** to regioisomeric 11,12-epoxyester **2b**.

**Figure 9:**
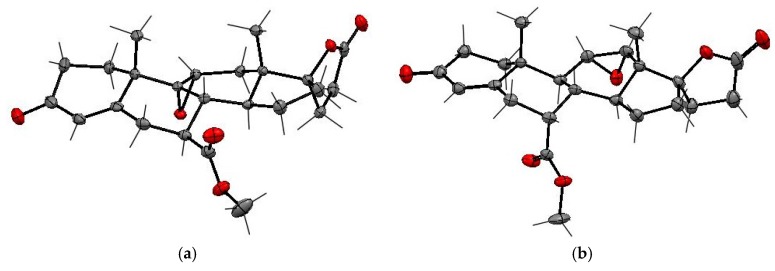
Molecular structure of the isomeric epoxides: (**a**) (7β,11α,17α)-9,11-epoxy-**2a** and (**b**) (7α,11α,12α,17α)-11,12-epoxy-**2b**.

The [M + Na]^+^ values, *m*/*z* 437.1941 and 437.1942, obtained for the two isomeric epoxyesters **2** and **2b** correspond to C_24_H_30_O_6_Na. The [M + H]^+^ value, *m*/*z* 415.2113, obtained for the third isomeric epoxyester **2a** corresponds to C_24_H_31_O_6_. The NMR structure assignments of the isomeric (7β,11α,17α)-9,11-epoxyester **2a** and (7α,11α,12α,17α)-11,12-epoxyester **2b** were confirmed by X-ray analysis (Figure 9 and Table 9). The SCXRD structures confirmed the presence of the 9α,11α-epoxide ring in **2a** and 11α,12α-epoxide ring in **2b**. The carbomethoxy group is situated at the C-7α position in **2b** and at the C-7β position in **2a**.

## 3. Experimental Section

### 3.1. General Information

11α-Hydroxy-7α-(methoxycarbonyl)-3-oxo-17α-pregn-4-ene-21,17-carbolactone (**12**, 98%) was manufactured by Hangzhou Pharma Chemicals Ltd. (Hangzhou, China) according to synthetic procedure described by Ng et al. [9] (Scheme 2). Sulfuryl chloride (97%), imidazole (99%), 2,2,2-trichloroacetamide (99%), disodium hydrogen phosphate (≥99%), hydrogen peroxide (35% in H_2_O) were purchased from Sigma-Aldrich (Munich, Germany) and CHEMPUR (Piekary Śląskie, Poland) chemical companies. Deionized water was prepared using a MilliQ plus purification system (Millipore, Bradford, PA, USA). Potassium bromide (FT-IR grade) and deuterated chloroform were purchased from Merck KGaA (Darmstad, Germany). The course of all reactions and the purity of products were checked by thin-layer chromatography (TLC). Analytical TLC was performed on silica gel DC-Alufolien Kieselgel 60 F_254_ (Merck KGaA), with mixtures of toluene, ethyl acetate, acetone, dichloromethane and acetonitrile, in various ratios as developing systems. Compounds were detected by spraying the plates with 1% Ce(SO_4_)_2_/2%H_3_[P(Mo_3_O_10_)_4_] in 10% H_2_SO_4_ followed by heating to 120 °C. Column chromatography was carried out on silica gel (Kieselgel 60, 40–63 μm, 230–400 mesh, Merck) with mixtures of ethyl acetate, toluene, acetone, acetonitrile and dichloromethane in varying ratios as eluents.

### 3.2. Optical Rotation

Optical rotations were measured with a Perkin Elmer 341 automatic polarimeter (Perkin Elmer, Norwalk, CT, USA) in CH_2_Cl_2_ solutions as the solvents with percent concentrations.

### 3.3. Melting Point

Melting points were determined with a MEL-TEMP II capillary melting point apparatus (Laboratory Devices, Holliston, MA, USA).

### 3.4. FT-IR Spectroscopy

FT-IR spectra were taken for KBr pellets on a Nicolet Impact 410 FT-IR spectrophotometer (Thermo Fisher Scientific, Waltham, MA, USA).

### 3.5. NMR Spectroscopy

The NMR spectra of all the compounds were measured in CDCl_3_ solutions with a Varian VNMRS-600 (600 MHz for ^1^H-NMR and 150 MHz for ^13^C-NMR; Varian Inc., Palo Alto, CA, USA) at temperature 298 K using TMS as internal standard. All ^1^H-/^13^C-NMR resonance signals were assigned using results of 2D experiments [g-COSY (^1^H-^1^H), g-HSQC (^1^H-^13^C) and g-HMBC (^1^H-^13^C)] in gradient versions. The ^1^H-NMR chemical shifts were determined as centres of the correlation spots in the ^1^H domain of the 2D ^1^H-^13^C HSQC experiments. Relative configuration and stereochemistry at the C-7 and other atoms was established on the basis of 1D and 2D NOESY spectra. Concentration of all solutions used for measurements was about 20–30 mg of compounds in 0.6 mL of solvent.

### 3.6. Mass Spectrometry

HRMS spectra were recorded on an AMD 604 Inectra GmbH (AMD Inectra GmbH, Harpstedt, Germany) and a Mariner PE Biosystem ESI-TOF (PerSeptive Biosystems/Applied Biosystems, Waltham, MA, USA) spectrometers.

### 3.7. X-ray Analysis

The X-ray diffraction data for **2b**, **7**, **7b**, **12**, **12a** and **13** were collected using a KM4CCD-axis diffractometer (Kuma Diffraction, Wrocław, Poland) with graphite-monochromated MoK radiation and equipped with an nitrogen gas-flow apparatus (Oxford Cryosystems, Oxford, UK). The data were corrected for Lorentz and polarization effects. The multi-scan absorption correction was applied for **12**, **12a** and **13**. Data reduction and analysis were carried out with the Oxford Diffraction Ltd. (Wrocław, Poland) suit of programs [29,30]. The structures were solved by direct methods approach using the SHELXS97 [31] program and refined with the SHELXL97 [32]. The X-ray diffraction data for **2a** and **7b** were collected using the Kappa APEX II Ultra (Bruker, Billerica, MA, USA) controlled by APEX II software [33], equipped with MoKα rotating anode X-ray source (λ = 0.71073 Å, 50.0 kV, 22.0 mA) monochromatized by multi-layer optics and APEX-II CCD detector. The experiments were carried out at 100 K using the Oxford Cryostream cooling device. Indexing, integration and initial scaling were performed with SAINT [34] and SADABS [35] software. The structures were solved by direct methods approach using the SHELXS97 [31] program and refined with the SHELXL97 [32]. Multi-scan absorption correction has been applied in the scaling procedure. Crystal data and refinement details for **2b**, **7**, **7a**–**b**, **12**, **12a** and **13** are listed in Table 3, Table 5, Table 6 and Table 9. The deposition numbers CCDC 1502629 (**2a**), CCDC 1565292 (**2b**), CCDC 1565294 (**7**), CCDC 1565293 (**7a**), CCDC 1502628 (**7b**), CCDC 1502627 (**12**), CCDC 1502631 (**12a**) and CCDC 1502630 (**13**) contain the supplementary crystallographic data for this paper. These data can be obtained free of charge from via http://www.ccdc.cam.ac.uk/conts/retrieving.html (or from the CCDC, 12 Union Road, Cambridge CB2 1EZ, UK; Fax: +44-1223-336-033; E-mail: deposit@ccdc.cam.ac.uk).

### 3.8. Syntheses

#### 3.8.1. 11α-Hydroxy-7β-(methoxycarbonyl)-3-oxo-17α-pregn-4-ene-21,17-carbolactone (**12a**, Scheme 3)

11-Hydroxyester (7α,11α,17α)-**12** (5.0 g, 12.0 mmol) was added to a suspension of MeONa (1.30 g, 24.0 mmol) in anhydrous MeOH (25 mL) and then refluxed for 24 h. The mixture was cooled to room temperature and 3 M aqueous HCl solution (150 mL) was added. The product was extracted with CH_2_Cl_2_ (3 × 25 mL). The combined organic phases were dried over anhydrous Na_2_SO_4_, filtered and concentrated under reduced pressure to give a mixture of epimers **12** and **12a** as a yellowish white foam. The crude product was purified by column chromatography over silica gel with 10–50% MeCN/PhCH_3_ gradient elution to give (7β,11α,17α)-**12a** (2.45 g, 49% yield) as a white solid, a mixture of **12** and **12a** (0.56 g) and the (7α,11α,17α)-**12** (1.27 g).

(7β,11α,17α)-**12a**: *R*_f_ = 0.40 for 60% MeCN/PhCH_3_. M.p. 250–252 °C. ${\left[\alpha \right]}_{D}^{20}$ = +21.49 (*c* 1.0, CHCl_3_). FT-IR (pellets, KBr) ν (cm^−1^): 3511, 2978, 2940, 2872, 2832, 1775, 1736, 1652, 1608, 1482, 1447, 1417, 1361, 1300, 1275, 1170, 1107, 1047, 1003, 914, 871, 789, 742, 672, 596, 509, 470. HRMS (ESI): calcd. for C_24_H_32_O_6_Na [M + Na]^+^ 439.20911, found 439.2110. ^1^H- and ^13^C-NMR spectra, see Table 2.

(7α,11α,17α)-**12**: *R*_f_ = 0.33 for 60% MeCN/PhCH_3_. M.p. 228–229 °C (lit. 229–230 °C [24]). ${\left[\alpha \right]}_{D}^{20}$ = +40.68 (*c* 1.0, CHCl_3_) (lit. ${\left[\alpha \right]}_{D}^{20}$ = +18.9 (*c* 0.0027, CHCl_3_) [24]). FT-IR (pellets, KBr) ν (cm^−1^): 3422, 3035, 2985, 2954, 2933, 2897, 2877, 1759, 1719, 1662, 1617, 1469, 1443, 1376, 1269, 1234, 1211, 1181, 1144, 1059, 1017, 999, 926, 866, 852, 787, 677, 606, 533, 514, 466. HRMS (ESI): calcd. for C_24_H_32_O_6_Na [M + Na]^+^ 439.20911, found 439.2080. ^1^H- and ^13^C-NMR spectra, see Table 2.

#### 3.8.2. Purification of the Commercial (7α,11α,17α)-11-hydroxyester **12** and Isolation of the (7β,11α,17α)-11-hydroxyester **12a** Impurity

The commercial 11-hydroxyester (7α,11α,17α)-**12** (32.5 g) was purified by recrystallization from a mixture of AcOEt/MeCN/CH_2_Cl_2_ (8:4:1) to afford the pure **12** (13.98 g, 43% yield) as a white crystals. The recrystallization mother liquors of **12**, containing a mixture of epimers **12** and **12a**, were subjected to column chromatography over silica gel with 10–50% MeCN/PhCH_3_ gradient elution to give (7β,11α,17α)-**12a** (0.91 g) as a white solid, a mixture of **12** and **12a** (14.4 g) and (7α,11α,17α)-**12** (2.67 g). The characterization data of **12** and **12a** were identical in all aspects with those obtained in experiment 3.8.1.

#### 3.8.3. 9,11β-dichloro-7α-(methoxycarbonyl)-3-oxo-17α-pregn-4-ene-21,17-carbolactone (**13**, Scheme 4)

A solution of (7α,17α)-9(11)-enester **7** (2.5 g, 6.27 mmol) in anhydrous pyridine (5.1 mL, 62.7 mmol) and chlorobenzene (25 mL) was cooled to 0 °C. SO_2_Cl_2_ (1.0 mL, 12.54 mmol) was added dropwise and the mixture was stirred for 30 min at 0 °C. The cooling bath was removed and the stirring was continued for 1 h at room temperature. The reaction mixture was diluted with H_2_O (100 mL) and the product was extracted with CH_2_Cl_2_ (3 × 50 mL).

The combined organic extracts were washed with aqueous saturated NaHCO_3_ solution (150 mL) followed by H_2_O (150 mL), dried over anhydrous Na_2_SO_4_, filtered and concentrated under reduced pressure to give a brown foam. The crude product was purified by column chromatography over silica gel with 5–30% AcOEt/CH_2_Cl_2_ gradient elution to afford 9,11-dichloro impurity **13** (1.21 g, 41% yield) as white crystals. *R*_f_ = 0.27 for 15% AcOEt/CH_2_Cl_2_. M.p. 230–231 °C. ${\left[\alpha \right]}_{D}^{20}$ = +123.30 (*c* 1.0, CHCl_3_). FT-IR (pellets, KBr) ν (cm^−1^): 3020, 2987, 2949, 2901, 2880, 1765, 1717, 1657, 1616, 1424, 1361, 1343, 1243, 1213, 1187, 1159, 1103, 1036, 1014, 965, 916, 868, 777, 663, 641, 601, 511. HRMS (ESI): calcd. for C_24_H_30_O_5_NaCl_2_ [M + Na]^+^ 491.13625, found 491.1386. ^1^H- and ^13^C-NMR spectra, see Table 7.

#### 3.8.4. 3-Oxo-17α-pregn-4-ene-7α,9:21,17-dicarbolactone (**14**, Imp. A, Scheme 5)

A suspension of 11-hydroxyester (7α,11α,17α)-**12** (5.0 g, 12.0 mmol) in anhydrous pyridine (25 mL) was cooled to 5 °C and MsCl (1.1 mL, 14.4 mmol) was added dropwise. A mixture was stirred at room temperature for 2 h followed by pyridine evaporation under reduced pressure. The residue was suspended in CH_2_Cl_2_ (25 mL), washed subsequently with aqueous 1 M HCl solution (3 × 25 mL), H_2_O (25 mL) and brine (25 mL). The organic phase was dried over anhydrous Na_2_SO_4_, filtered and evaporated under reduced pressure.

The crude product was purified by column chromatography over silica gel with 0.5–2% MeOH/CH_2_Cl_2_ gradient elution to afford mesylate (5.04 g, 85% yield) as white crystals. A mixture of mesylate (2.5 g, 5.05 mmol), AcONa (4.14 g, 50.5 mmol), 80% AcOH (15 mL) and H_2_O (5 mL) was heated at 70 °C for 3 h followed by AcOH evaporation under reduced pressure. Saturated aqueous Na_2_CO_3_ solution (25 mL) was added to the residue and the product was extracted with CH_2_Cl_2_ (3 × 25 mL). The combined organic phases were washed with brine (100 mL), dried over anhydrous Na_2_SO_4_, filtered and concentrated under reduced pressure. The crude product was purified by column chromatography over silica gel with 5–30% AcOEt/CH_2_Cl_2_ gradient elution to afford 7α,9:21,17-dicarbolactone 14 (1.61 g, 83% yield) as a white solid. *R*_f_ = 0.20 for 15% AcOEt/CH_2_Cl_2_. M.p. 244–245 °C. ${\left[\alpha \right]}_{D}^{20}$ = −2.41 (*c* 1.0, CHCl_3_). FT-IR (pellets, KBr) ν (cm^−1^): 2964, 2875, 1770, 1674, 1627, 1452, 1383, 1344, 1268, 1196, 1177, 1157, 1110, 1047, 971, 914, 874, 814, 722, 657, 593, 509. HRMS (ESI): calcd. for C_23_H_28_O_5_Na [M + Na]^+^ 407.18289, found 407.1847. ^1^H- and ^13^C-NMR spectra, see Table 7.

#### 3.8.5. 7α-(Methoxycarbonyl)-3-oxo-17α-pregna-4,9(11)-diene-21,17-carbolactone (**7**, Imp. C, Scheme 6)

Imidazole (13.08 g, 192.08 mmol) was added to a solution of 11-hydroxyester (7α,11α,17α)-**12** (20.0 g, 48.02 mmol) in THF (150 mL) and cooled to −10 °C. SO_2_Cl_2_ (8.2 mL, 100.84 mmol) was added dropwise and the mixture was stirred for 30 min. at −10 °C. The cooling bath was then removed and the stirring was continued for 1 h at room temperature. The reaction mixture was diluted with H_2_O (100 mL) and the product was extracted with CH_2_Cl_2_ (3 × 50 mL). The combined organic extracts were washed with aqueous saturated NaHCO_3_ solution (100 mL), dried over anhydrous Na_2_SO_4_, filtered and concentrated under reduced pressure to give a yellowish white foam.

The crude product was recrystallized from ethanol and a mixture of dichloromethane/diethyl ether to give (7α,17α)-9(11)-enester 7 (13.58 g, 71% yield). *R*_f_ = 0.17 for 15% AcOEt/CH_2_Cl_2_. M.p. 204–206 °C (lit. 205–206 °C [17]). ${\left[\alpha \right]}_{D}^{20}$ = +2.75 (*c* 1.0, CHCl_3_). FTIR (KBr) ν (cm^−1^): 3052, 2967, 2908, 2875, 1774, 1732, 1666, 1622, 1463, 1438, 1421, 1378, 1332, 1268, 1166, 1090, 1012, 966, 920, 868, 834, 770, 665, 614, 531, 475. HRMS (ESI): calcd. for C_24_H_30_O_5_Na [M + Na]^+^ 421.19855, found 421.1986. ^1^H- and ^13^C-NMR spectra, see Table 4.

#### 3.8.6. 7β-(methoxycarbonyl)-3-oxo-17α-pregna-4,9(11)-diene-21,17-carbolactone (**7a**, Scheme 7 and Scheme 8)

##### Method 1

The (7α,17α)-9(11)-enester **7** (3.2 g, 8.03 mmol) was added to a suspension of MeONa (1.52 g, 28.11 mmol) in anhydrous MeOH (20 mL) and then refluxed for 24 h. The mixture was cooled to room temperature and aqueous 3 M HCl solution (100 mL) was added. The product was extracted with CH_2_Cl_2_ (3 × 25 mL). The combined organic phases were dried over anhydrous Na_2_SO_4_, filtered and concentrated under reduced pressure to give a brownish foam.

The crude mixture of products was purified by column chromatography over silica gel with 5–20% AcOEt/CH_2_Cl_2_ gradient elution to give (7β,17α)-9(11)-enester **7a** (1.18 g, 37% yield) as a white solid, a mixture of epimers **7** and **7a** (0.78 g) and (7α,17α)-9(11)-enester **7** (1.12 g).

(7β,17α)-9(11)-enester **7a**: *R*_f_ = 0.34 for 15% AcOEt/CH_2_Cl_2_. M.p. 167–168 °C. ${\left[\alpha \right]}_{D}^{20}$ = +0.50 (*c* 1.0, CHCl_3_). FT-IR (pellets, KBr) ν (cm^−1^): 3053, 2970, 2883, 2847, 1773, 1731, 1671, 1618, 1435, 1369, 1299, 1265, 1188, 1159, 1090, 1017, 990, 910, 868, 816, 792, 769, 665, 602, 521, 480. HRMS (ESI): calcd. for C_24_H_30_O_5_Na [M + Na]^+^ 421.19855, found 421.2006. ^1^H- and ^13^C-NMR spectra, see Table 4. The characterization data of **7** were identical in all aspects with those obtained in experiment 3.8.5.

##### Method 2

Imidazole (1.31 g, 19.20 mmol) was added to a solution of 11-hydroxyester (7β,11α,17α)-**12a** (2.0 g, 4.80 mmol) in THF (25 mL) and cooled to −20 °C. SO_2_Cl_2_ (0.8 mL, 10.08 mmol) was added dropwise and the mixture was stirred for 30 min. at −20 °C. The cooling bath was then removed and the stirring was continued for 1 h at room temperature. The reaction mixture was diluted with H_2_O (10 mL) and the product was extracted with CH_2_Cl_2_ (3 × 25 mL).

The combined organic extracts were washed with aqueous saturated NaHCO_3_ solution (50 mL), dried over anhydrous Na_2_SO_4_, filtered and concentrated under reduced pressure to give a yellowish white foam. The crude product was purified by column chromatography over silica gel with 5–20% AcOEt/CH_2_Cl_2_ gradient elution to give (7β,17α)-9(11)-enester **7a** (1.62 g, 85% yield). The characterization data of **7a** were identical in all aspects with those obtained in Method 1.

#### 3.8.7. 7α-(Methoxycarbonyl)-3-oxo-17α-pregna-4,11(12)-diene-21,17-carbolactone (**7b**)

The recrystallization mother liquors of **7** (9.81 g) were chromatogaphed on silica gel using varying mixtures of ethyl acetate, dichloromethane, acetonitrile and toluene. Early cuts of the 5–30% AcOEt/CH_2_Cl_2_ gradient elution afforded (7β,17α)-9(11)-enester **7a** (0.48 g) and (7α,17α)-11(12)-enester **7b** (0.73 g) as white crystalline products. An analytical sample of **7b** was prepared by a recrystallization from ethanol. Succeeding cuts of 5–30% AcOEt/CH_2_Cl_2_ gradient elution gave (7α,11β,17α)-9,11-dichloro impurity **14** (0.84 g), 7,9:21,17-dilactone **13** (1.16 g) and (7α,17α)-9(11)-enester 7 (2.75 g) as white crystalline products. Finally, crystalline 11-hydroxyesters (7β,11α,17α)-**12a** (0.17 g) and (7α,11α,17α)-**12** (0.33 g) were obtained on further gradient elution with 10–50% MeCN/PhCH_3_. (7α,17α)-11(12)-enester **7b**: *R*_f_ = 0.29 for 15% AcOEt/CH_2_Cl_2_. M.p. 232–234 °C. ${\left[\alpha \right]}_{D}^{20}$ = +33.42 (*c* 1.0, CHCl_3_). FT-IR (pellets, KBr) ν (cm^−1^): 3028, 2971, 2949, 2878, 1775, 1723, 1664, 1616, 1465, 1438, 1380, 1334, 1267, 1195, 1172, 1048, 1013, 918, 868, 788, 716, 669, 525, 454. HRMS (ESI): calcd. for C_24_H_30_O_5_Na [M + Na]^+^ 421.1991, found 421.1988. ^1^H- and ^13^C-NMR spectra, see Table 4.

The characterization data of **7**, **7a**, **12**, **12a**, **13** and **14** were identical in all aspects with those obtained in experiments 3.8.1–3.8.6, respectively.

#### 3.8.8. 11α,12α-Epoxy-7α-(methoxycarbonyl)-3-oxo-17α-pregn-4-ene-21,17-carbolactone (**2b**, Imp. B, Scheme 9)

2,2,2-Trichloroacetamide (0.52 g, 3.17 mmol) and Na_2_HPO_4_ (0.35 g, 2.48 mmol) were added to a stirred solution of (7α,17α)-11(12)-enester **7b** (0.55 g, 1.38 mmol) in CH_2_Cl_2_ (15 mL). The mixture was cooled to 15 °C and H_2_O_2_ (35%, 8.4 mL) was added dropwise. After being stirred at room temperature for 48 h, the mixture was diluted with H_2_O (15 mL) and the product was extracted with CH_2_Cl_2_ (3 × 10 mL). The combined organic extracts were washed in succession with 3% aqueous Na_2_SO_3_ solution (25 mL), 1 M aqueous NaOH solution (20 mL), 1 M aqueous HCl solution (20 mL) and brine (25 mL).

The organic phase was dried over anhydrous Na_2_SO_4_, filtered and concentrated under reduced pressure to give a yellowish white foam. The crude 11,12-epoxide was purified by column chromatography over silica gel with 1–10% Me_2_CO/CH_2_Cl_2_ gradient elution to give 2b (0.34 g, 60% yield) as a white solid. *R*_f_ = 0.37 for 10% Me_2_CO/CH_2_Cl_2_. M.p. 209–210 °C. ${\left[\alpha \right]}_{D}^{20}$ = +24.63 (*c* 1.0, CH_2_Cl_2_). FT-IR (pellets, KBr) ν (cm^−1^): 3531, 3430, 3024, 2952, 2880, 1780, 1725, 1664, 1614, 1463, 1437, 1421, 1404, 1381, 1335, 1295, 1268, 1237, 1194, 1174, 1072, 1034, 1011, 977, 962, 920, 880, 836, 784, 664, 629, 605, 524, 497, 454. HRMS (ESI): calcd. for C_24_H_30_O_6_Na [M + Na]^+^ 437.1940, found 437.1942. ^1^H- and ^13^C-NMR spectra, see Table 8.

#### 3.8.9. 9,11α-Epoxy-7β-(methoxycarbonyl)-3-oxo-17α-pregn-4-ene-21,17-carbolactone (**2a**, Imp. E, Scheme 10 and Scheme 11).

##### Method 1

Eplerenone (3.1 g, 7.48 mmol) was added to a suspension of MeONa (1.41 g, 26.17 mmol) in anhydrous MeOH (25 mL) and then refluxed for 24 h. The mixture was cooled to room temperature and 3 M aqueous HCl solution (150 mL) was added. The product was extracted with CH_2_Cl_2_ (3 × 25 mL).

The combined organic phases were dried over anhydrous Na_2_SO_4_, filtered and concentrated under reduced pressure to give a yellowish white solid. The crude mixture of epimers **2** and **2a** was purified by column chromatography over silica gel with 1–10% Me_2_CO/CH_2_Cl_2_ gradient elution to give (7β,11α,17α)-9,11-epoxyester **2a** (1.52 g, 49% yield) as a white solid, a mixture of **2** and **2a** (0.55 g) and (7α,11α,17α)-9,11-epoxyester **2** (0.77 g).

(7β,11α,17α)-9,11-epoxyester 2a: *R*_f_ = 0.43 for 10% Me_2_CO/CH_2_Cl_2_. M.p. 249–251 °C (lit. 254–258 °C [9]). ${\left[\alpha \right]}_{D}^{20}$ = +12.53 (*c* 1.0, CHCl_3_). FT-IR (pellets, KBr) ν (cm^−1^): 3030, 2972, 2953, 2929, 2883, 1774, 1734, 1674, 1619, 1436, 1384, 1296, 1269, 1195, 1165, 1080, 1013, 905, 866, 797, 659, 576, 521, 414. HRMS (ESI): calcd. for C_24_H_31_O_6_ [M + H]^+^ 415.21152, found 415.2113. ^1^H- and ^13^C-NMR spectra, see Table 8. The characterization data of **2** were identical in all aspects with those obtained in experiment 3.8.10.

##### Method 2

2,2,2-Trichloroacetamide (0.98 g, 6.02 mmol) and Na_2_HPO_4_ (1.21 g, 8.53 mmol) were added to a stirred solution of (7β,17α)-11(12)-enester **7a** (2.0 g, 5.02 mmol) in CH_2_Cl_2_ (50 mL). The mixture was cooled to 15 °C and H_2_O_2_ (35%, 4.2 mL) was added dropwise. After being stirred at room temperature for 48 h, the mixture was diluted with H_2_O (20 mL) and the product was extracted with CH_2_Cl_2_ (3 × 25 mL). The combined organic extracts were washed in succession with 3% aqueous Na_2_SO_3_ solution (50 mL), 1 M aqueous NaOH solution (50 mL), 1 M aqueous HCl solution (50 mL) and brine (50 mL).

The organic phase was dried over anhydrous Na_2_SO_4_, filtered and concentrated under reduced pressure to give a yellowish white foam. The crude product was purified by column chromatography over silica gel with 1–10% Me_2_CO/CH_2_Cl_2_ gradient elution to give the (7β,11α,17α)-9,11-epoxyester **2a** (1.44 g, 69% yield) as a white solid. The characterization data of **2a** were identical in all aspects with those obtained in Method 1.

#### 3.8.10. 9,11α-Epoxy-7α-(methoxycarbonyl)-3-oxo-17α-pregn-4-ene-21,17-carbolactone (eplerenone, **2**) (without purification of the enester **7**, Scheme 12)

Imidazole (6.53 g, 96.04 mmol) was added to a solution of 11-hydroxyester (7α,11α,17α)-**12** (10.0 g, 24.01 mmol) in THF (100 mL) and cooled to −10 °C. SO_2_Cl_2_ (4.1 mL, 50.42 mmol) was added dropwise and the mixture was stirred for 30 min. at −10 °C. The cooling bath was then removed and the stirring was continued for 1 h at room temperature.

The reaction mixture was diluted with H_2_O (100 mL) and the product was extracted with CH_2_Cl_2_ (3 × 50 mL). The combined organic extracts were washed with aqueous saturated NaHCO_3_ solution (100 mL), dried over anhydrous Na_2_SO_4_, filtered and concentrated under reduced pressure to give a yellowish white foam. The crude (7α,17α)-9(11)-enester **7** was dissolved in CH_2_Cl_2_ (150 mL), followed by 2,2,2-trichloroacetamide (4.68 g, 28.81 mmol) and Na_2_HPO_4_ (5.79 g, 40.82 mmol) addition. The mixture was cooled to 15 °C and H_2_O_2_ (35%, 40.0 mL) was added under vigorous stirring. The cooling bath was removed. After being stirred at room temperature for 18 h, H_2_O (50 mL) and crushed ice (50 g) were added to the reaction mixture and the layers were separated. The organic phase was washed with NaOH (0.5 M, 100 mL), HCl (0.5 N, 100 mL), brine (100 mL), dried over anhydrous Na_2_SO_4_ and filtered under reduced pressure to give a yellowish white foam. The crude product was recrystallized twice from ethanol and from 2-butanone to give the pharmaceutical grade (7α,11α,17α)-9,11-epoxyester **2** (5.20 g, 52% yield) as a white crystals. *R*_f_ = 0.27 for 10% Me_2_CO/CH_2_Cl_2_. M.p. 241–243 °C (lit. 240–242 °C [17]). ${\left[\alpha \right]}_{D}^{20}$ = +1.55 (*c* 1.0, CHCl_3_). FTIR (pellets, KBr) ν (cm^−1^): 2997, 2969, 2950, 2877, 1778, 1725, 1656, 1619, 1460, 1444, 1427, 1382, 1293, 1273, 1183, 1160, 1081, 1018, 985, 919, 848, 797, 722, 694, 656, 543, 520, 460. HRMS (ESI): calcd. for C_24_H_30_O_6_Na [M + Na]^+^ 437.1940, found 437.1941. ^1^H- and ^13^C-NMR spectra, see Table 8. The recrystallization mother liquors of **2** (4.1 g) were chromatographed on silica gel using varying mixtures of acetone and dichloromethane. Early cuts of the 1–10% Me_2_CO/CH_2_Cl_2_ gradient elution afforded the (7α,11β,17α)-9,11-dichloro impurity **13** (0.46 g) and the (7β,11α,17α)-9,11-epoxy ester **2a** (0.19 g). Succeeding cuts of the 1–10% Me_2_CO/CH_2_Cl_2_ gradient elution gave the 7,9:21,17-dilactone **14** (0.85 g) and the (7α,11α,17α)-11,12-epoxy ester **2b** (0.26 g). The characterization data of **2a**, **2b**, **13** and **14** were identical in all aspects with those obtained in experiments 3.8.3, 3.8.4, 3.8.8 and 3.8.9, respectively.

## 4. Conclusions

Two new process-related impurities of the antihypertensive drug eplerenone (**2**) were synthesized and fully characterized. The impurities were identified as 11α-hydroxy-7β-(methoxycarbonyl)-3-oxo-17α-pregn-4-ene-21,17-carbolactone (**12a**) and 9,11β-dichloro-7α-(methoxycarbonyl)-3-oxo-17α-pregn-4-ene-21,17-carbolactone (**13**). Additionally, seven other eplerenone impurities poorly described in the literature, including four impurities A, B, C and E listed in the European Pharmacopoeia 8.4, were isolated and/or synthesized and fully characterized by IR, NMR, HRMS/ESI and SCXRD. All the impurities resulted from side reactions taking place on the steroid rings B and C of the starting (7α,11α,17α)-11-hydroxyester **12** and the key intermediate (7α,17α)-9(11)-enester **7**, including epimerization of the C-7 asymmetric center, oxidation, dehydration, chlorination and lactonization. The full identification and characterization of the impurities should be useful for the quality control and the validation of the analytical methods in the manufacture of eplerenone.

## Figures and Tables

**Scheme 3:**
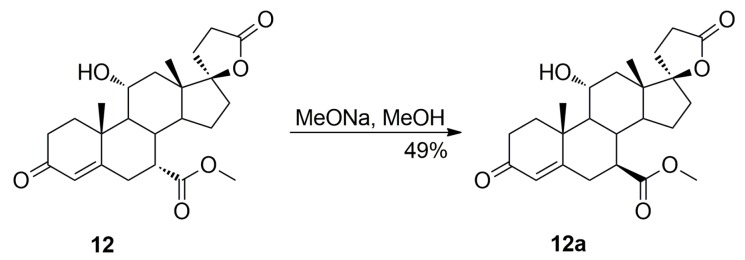
Synthesis of the 11-hydroxyester (7β,11α,17α)-**12a**.

**Scheme 4:**
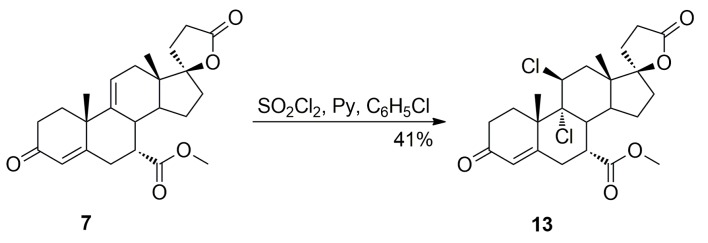
Synthesis of the (7α,11β,17α)-9,11-dichloro derivative **13**.

**Scheme 5:**
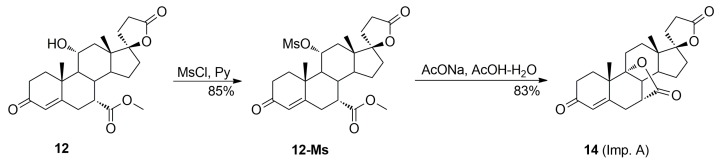
Synthesis of the 7,9:21,17-dilactone **14** (Imp. A).

**Scheme 6:**
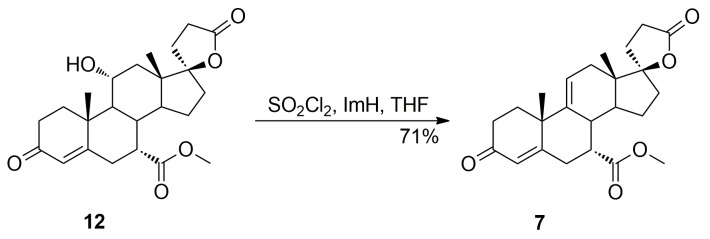
Synthesis of the (7α,17α)-9(11)-enester **7**.

**Scheme 7:**
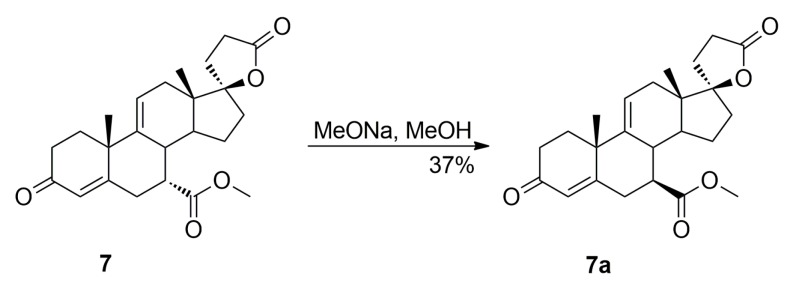
Synthesis of the (7β,17α)-9(11)-enester **7a**.

**Scheme 8:**
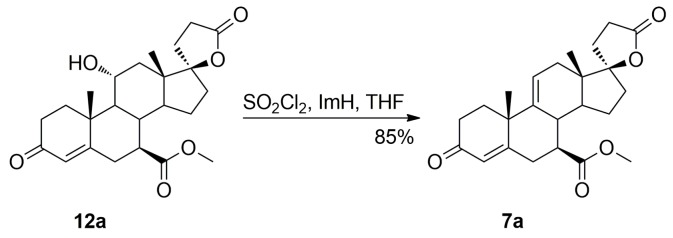
Synthesis of the (7β,17α)-9(11)-enester **7a**.

**Scheme 9:**
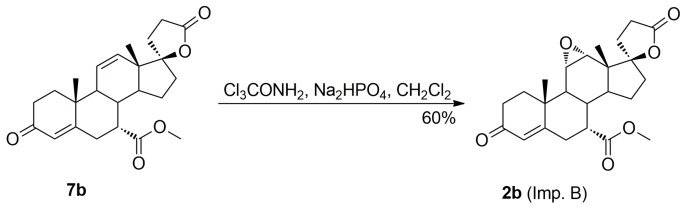
Synthesis of the (7α,11α,12α,17α)-11,12-epoxyester **2b** (Imp. B).

**Scheme 10:**
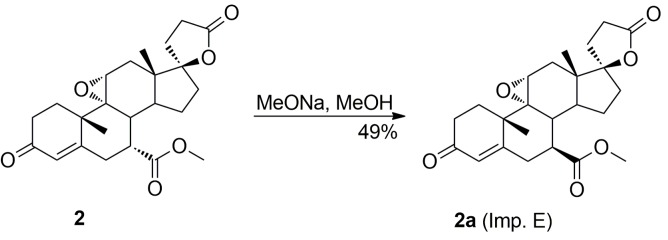
Synthesis of the (7β,11α,17α)-9,11-epoxyester (**2a**, Imp. E).

**Scheme 11:**
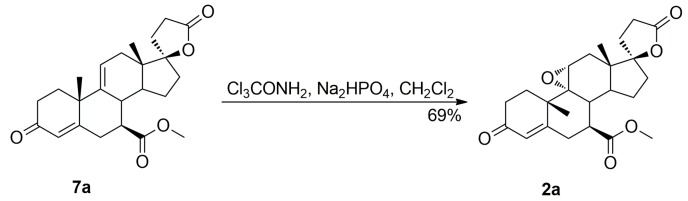
Synthesis of the (7β,11α,17α)-9,11-epoxyester (**2a**, Imp. E).

**Scheme 12:**
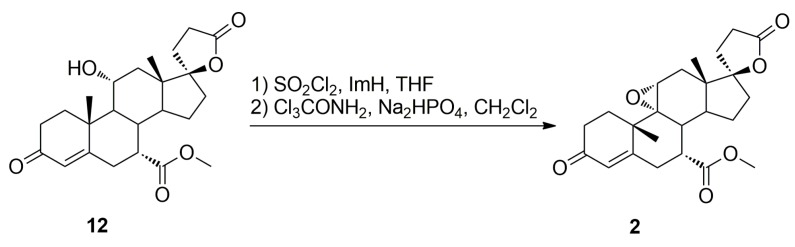
Synthesis of the (7α,11α,12α,17α)-9,11-epoxyester **2**.

**Table 1:** Structures of the process-related impurities of eplerenone (**2**).

Chemical Structure	Name
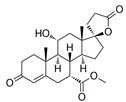	11α-hydroxy-7α-(methoxycarbonyl)-3-oxo-17α-pregn-4-ene-21,17-carbolactone
**12**	
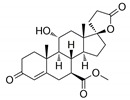	11α-hydroxy-7β-(methoxycarbonyl)-3-oxo-17α-pregn-4-ene-21,17-carbolactone
**12a**	
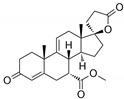	7α-(methoxycarbonyl)-3-oxo-17α-pregna-4,9(11)-diene-21,17-carbolactone
**7** (Imp. C) ^1^	
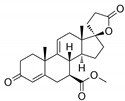	7β-(methoxycarbonyl)-3-oxo-17α-pregna-4,9(11)-diene-21,17-carbolactone
**7a**	
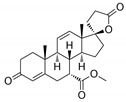	7α-(methoxycarbonyl)-3-oxo-17α-pregna-4,11(12)-diene-21,17-carbolactone
**7b**	
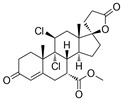	9,11β-dichloro-7α-(methoxycarbonyl)-3-oxo-17α-pregn-4-ene-21,17-carbolactone
**13**	
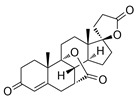	3-oxo-17α-pregn-4-ene-7α,9:21,17-dicarbolactone
**14** (Imp. A) ^1^	
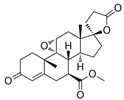	9,11α-epoxy-7β-(methoxycarbonyl)-3-oxo-17α-pregn-4-ene-21,17-carbolactone
**2a** (Imp. E) ^1^	
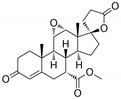	11α,12α-epoxy-7α-(methoxycarbonyl)-3-oxo-17α-pregn-4-ene-21,17-carbolactone
**2b** (Imp. B) ^1^	

^1^ Impurities listed in the European Pharmacopoeia 8.4 [23].

**Table 2:** The ^1^H- and ^13^C-NMR spectral data in CDCl_3_ for 11-hydroxyesters (7α,11α,17α)-**12** and (7β,11α,17α)-**12a**.

^1^H-NMR ^(1)^			^13^C-NMR		
Proton	12	12a	Carbon	12	12a
H-1a	2.02 m	2.07 m	C-1	37.3	37.1
H-1b	2.80 m	2.71 m	C-2	34.1	34.2
H-2a	2.34 m	2.33 m	C-3	199.5	199.9
H-2b	2.46 m	2.43 m	C-4	126.1	125.0
H-4	5.69 s	5.75 s	C-5	167.7	167.2
H-6a	2.52 m	2.37 m	C-6	36.3	37.3
H-6b	2.64 ddd (1.9, 5.8, 14.6)	2.62 m	C-7	42.4	49.0
H-7	2.81 m	2.31 m	C-8	37.0	37.0
H-8	1.79 m	2.14 m	C-9	52.6	57.4
H-9	1.93 m	1.22 t (10.4)	C-10	39.4	39.6
H-11	4.04 m	4.10 m	C-11	69.2	68.5
H-12a	1.42 m	1.42 m	C-12	43.4	43.5
H-12b	1.85 m	1.83 dd (4.6, 11.6)	C-13	46.2	46.6
H-14	1.65 m	1.37 m	C-14	45.3	48.7
H-15a	1.41 m	1.49 m	C-15	22.5	22.5
H-15b	1.73 m	1.49 m	C-16	35.4	35.6
H-16a	1.88 m	1.80 ddd (6.9, 8.8, 14.3 )	C-17	95.0	94.5
H-16b	2.26 ddd (3.6, 12.1, 14.5)	2.21 ddd (4.4, 11.4, 14.3)	C-18	15.4	16.1
H-18	1.01 s	1.03 s	C-19	18.4	18.4
H-19	1.36 s	1.40 s	C-20	31.2	31.4
H-20a	1.95 m	1.93 ddd (7.5, 9.4, 13.0)	C-21	29.1	29.2
H-20b	2.39 m	2.37 m	C-22	176.5	176.5
H-21a	2.50 m	2.49 ddd (11.6, 13.1, 17.7)	C-23	172.7	175.4
H-21b	2.57 m	2.56 ddd (6.2, 9.3, 17.7)	C-24	51.6	52.1
H-24	3.65 s	3.69 s			

^1^ Values in parentheses give δ, multiplicity and *J* in Hz.

**Table 3:** Summary of crystallographic data and structure refinement for the 11-hydroxyesters (7α,11α,17α)-**12** and (7β,11α,17α)-**12a**.

Identification Code	12	12a
Chemical formula	C_24_H_32_O_6_	C_24_H_32_O_6_
Molecular weight	416.50 g/mol	416.50 g/mol
Temperature	100(2) K	100(2) K
Wavelength	0.71073 Å	0.71073 Å
Crystal system, space group	Orthorhombic, P2_1_2_1_2_1_	Orthorhombic, P2_1_2_1_2_1_
Unit cell dimensions	a = 6.7560(2) Å	a = 12.2862(3) Å
	b = 16.1436(5) Å	b = 12.4532(3) Å
	c = 19.0947(6) Å	c = 13.3060(4) Å
	α = 90°	α = 90°
	β = 90 °	β = 90°
	γ = 90°	γ = 90°
Volume	2082.59(11) Å^3^	2035.85(9) Å^3^
*Z*, Calculated density	4, 1.328 mg/m^3^	4, 1.359 mg/m^3^
Absorption coefficient	0.094	0.096
*F*(000)	896	896
Crystal size	0.30 × 0.20 × 0.20 mm	0.30 × 0.20 × 0.10 mm
Theta range for data collection	2.13°–25.68°	2.26°–25.67°
Limiting indices	−8 ≤ *h* ≤ 8	−14 ≤ *h* ≤ 14
	−19 ≤ *k* ≤ 19	−15 ≤ *k* ≤ 15
	−23 ≤ *l* ≤ 23	−16 ≤ *l* ≤ 16
Reflections collected/unique	21,220/3973	11,318/3856
Absorption correction	semi-empirical from equivalents	semi-empirical from equivalents
Max. and min. transmission	0.9814 and 0.99723	0.9904 and 0.9716
Refinement method	full-matrix least-squares on *F*^2^	full-matrix least-squares on *F*^2^
Data/restraints/parameters	3973/0/275	3856/1/275
Goodness-of-fit on *F^2^*	1.156	1.041
Final *R*1/*wR2* indices [*I* > 2sigma(*I*)]	0.0360/0.0921	0.0311/0.0721
*R1/wR2* indices (all data)	0.0481/0.1132	0.0361/0.0756
Largest diff. peak and hole	0.191 and −0.341 e/Å^3^	0.172 and −0.212 e/Å^3^

**Table 4:** The ^1^H- and ^13^C-NMR spectral data for esters (7α,17α)-9(11)-ene **7**, (7β,17α)-9(11)-ene **7a** and (7α,17α)-11-ene **7b**.

^1^H-NMR ^(1)^				^13^C-NMR			
Proton	7	7a	7b	Carbon	7	7a	7b
H-1a	2.18 m	2.12 m	1.87 m	C-1	33.7	33.9	34.9
H-1b	2.24 m	2.12 m	2.15 m	C-2	34.2	34.2	33.7
H-2a	2.53 m	2.50 m	2.45 m	C-3	198.6	198.6	198.7
H-2b	2.53 m	2.50 m	2.45 m	C-4	125.7	124.6	126.8
H-4	5.71 d (2.0)	5.75 d (2.0)	5.81 s	C-5	166.6	165.5	166.3
H-6a	2.55 m	2.43 dd (3.8, 14.6)	2.67 m	C-6	35.7	36.2	35.6
H-6b	2.82 ddd (2.0, 5.3, 15.0)	2.85 ddd (2.0, 13.2, 14.6)	2.67 m	C-7	43.8	49.7	42.1
H-7	2.98 m	2.28 m	2.89 m	C-8	40.4	40.4	35.9
H-8	2.48 m	2.70 m	2.24 m	C-9	142.3	142.1	49.2
H-9	-	-	2.44 m	C-10	40.5	40.9	38.8
H-11	5.65 m	5.68 m	5.59 dd (2.1, 10.2)	C-11	119.0	120.4	126.5
H-12a	1.87 m	1.88 m	5.89 dd (2.9, 10.2)	C-12	32.9	32.7	133.8
H-12b	2.20 m	2.17 m	-	C-13	44.5	44.7	49.0
H-14	1.49 m	1.50 m	1.95 m	C-14	43.1	45.5	44.1
H-15a	1.49 m	1.54 m	1.57 m	C-15	23.3	23.8	21.8
H-15b	1.89 m	1.54 m	1.87 m	C-16	35.4	35.4	35.7
H-16a	1.87 m	1.81 m	1.95 m	C-17	95.1	94.5	93.9
H-16b	2.31 m	2.26 m	2.20 m	C-18	14.1	14.3	18.6
H-18	0.95 s	0.96 s	1.05 s	C-19	27.2	25.7	17.3
H-19	1.39 s	1.42 s	1.16 s	C-20	31.4	31.2	32.3
H-20a	1.92 m	1.93 m	2.00 m	C-21	29.2	29.1	28.7
H-20b	2.29 m	2.30 m	2.21 m	C-22	176.5	176.4	176.7
H-21a	2.50 m	2.51 m	2.61 m	C-23	172.6	174.9	173.0
H-21b	2.59 ddd (6.0, 9.4, 17.8)	2.60 m	2.53 m	C-24	51.4	52.1	51.6
H-24	3.59 s	3.72 s	3.66 s				

^1^ Values in parentheses give δ, multiplicity and *J* in Hz.

**Table 5:** Summary of crystallographic data and structure refinement for (7α,17α)-11(12)-enester **7b** and (7α,11β,17α)-9,11-dichloro derivative **13**.

Identification Code	7b	13
Chemical formula	C_24_H_30_O_5_	C_24_H_31_Cl_2_O_5_
Molecular weight	398.48 g/mol	470.39 g/mol
Temperature	100(2) K	100(2) K
Wavelength	0.71073 Å	0.71073 Å
Crystal system, space group	Orthorhombic, P2_1_2_1_2_1_	Orthorhombic, P2_1_2_1_2_1_
Unit cell dimensions	a = 6.2322(5) Å	a = 9.2383(4) Å
	b = 15.8874(13) Å	b = 14.2496(6) Å
	c = 20.5238(15) Å	c = 16.3898(7) Å
	α = 90°	α = 90°
	β = 90°	β = 90°
	γ = 90°	γ = 90°
Volume	2032.1(3) Å^3^	2157.59(16) Å^3^
*Z*, Calculated density	4, 1.302 mg/m^3^	4, 1.448 mg/m^3^
Absorbtion coefficient	0.090	0.336
*F*(000)	856	996
Crystal size	0.30 × 0.15 × 0.15 mm	0.30 × 0.10 × 0.10 mm
Theta range for data collection	2.36°–26.37°	1.89°–25.68°
Limiting indices	−7 ≤ *h* ≤ 7	−11 ≤ *h* ≤ 11
	−19 ≤ *k* ≤ 19	−17 ≤ *k* ≤ 17
	−25 ≤ *l* ≤ 25	−19 ≤ *l* ≤ 19
Reflections collected/unique	35,771/4149	21,917/4092
Absorption correction	semi-empirical from equivalents	semi-empirical from equivalents
Max. and min. transmission	0.9866 and 0.9735	0.9671 and 0.9058
Refinement method	full-matrix least-squares on *F*^2^	full-matrix least-squares on *F*^2^
Data/restraints/parameters	4149/0/265	4092/0/283
Goodness-of-fit on *F^2^*	1.017	1.024
Final *R*1/*wR2* indices [*I* > 2sigma(*I*)]	0.0319/0.0883	0.0368/0.0698
*R1*/*wR2* indices (all data)	0.0337/0.0903	0.0515/0.0749
Largest diff. peak and hole	0.295 and −0.135 e/Å^3^	0.231 and −0.244 e/Å^3^

**Table 6:** Summary of crystallographic data and structure refinement for the isomeric 9(11)-enesters (7α,17α)-**7** and (7β,17α)-**7a**.

Identification Code	7	7a
Chemical formula	C_24_H_30_O_5_	C_24_H_30_O_5_
Molecular weight	398.48 g/mol	398.48 g/mol
Temperature	100(2) K	100(2) K
Wavelength	0.71073 Å	0.71073 Å
Crystal system, space group	Orthorhomblic, *P*2_1_2_1_2_1_	Orthorhomblic, *P*2_1_2_1_2_1_
Unit cell dimensions	a = 6.296(2) Å	a = 12.099(3) Å
	b = 15.468(3) Å	b = 12.310(3) Å
	c = 21.088(3) Å	c = 13.497(3) Å
	α = 90°	α = 90°
	β = 90°	β = 90°
	γ = 90°	γ = 90°
Volume	2053.7(8) Å^3^	2010.2(8) Å^3^
*Z*, Calculated density	4, 1.289 mg/m^3^	4, 1.317 mg/m^3^
Absorbtion coefficient	0.089	0.091
*F*(000)	856	856
Crystal size	0.41 × 0.32 × 0.29 mm	0.60 × 0.39 × 0.31
Theta range for data collection	3.183°−28.719°	3.310°−36.896°
Limiting indices	−8 ≤ *h* ≤ 8	−15 ≤ *h* ≤ 20
	−19 ≤ *k* ≤ 16	−19 ≤ *k* ≤ 20
	−27 ≤ *l* ≤ 28	−22 ≤ *l* ≤ 22
Reflections collected/unique	14,340/4925	36,105/9538
Absorption correction	None	None
Max. and min. transmission	0.975 and 0.964	0.972 and 0.947
Refinement method	full-matrix least-squares on *F*^2^	full-matrix least-squares on *F*^2^
Data/restraints/parameters	4925/0/262	9538/0/262
Goodness-of-fit on *F^2^*	0.929	0.975
Final *R*1/*wR2* indices [*I* > 2sigma(*I*)]	0.0475/0.1011	0.0484/0.1205
*R1/wR2* indices (all data)	0.0673/0.1071	0.0622/0.1259
Largest diff. peak and hole	0.189/−0.220 e/Å^3^	0.453/−0.303 e/Å^3^

**Table 7:** The ^1^H- and ^13^C-NMR spectral data in CDCl_3_ for (7α,11β,17α)-9,11-dichloro derivative **13** and 7α,9:21,17-dicarbolactone **14**.

^1^H-NMR ^(1)^			^13^C-NMR		
Proton	13	14	Carbon	13	14
H-1a	2.27 m	1.88 m	C-1	29.6	29.6
H-1b	2.77 m	2.27 m	C-2	33.8	33.2
H-2a	2.50 m	2.49 m	C-3	197.8	197.1
H-2b	2.50 m	2.49 m	C-4	125.7	129.4
H-4	5.89 d (1.8)	5.86 d (2.0)	C-5	163.7	161.5
H-6a	2.60 m	2.65 dd (4.0, 15.4)	C-6	32.4	34.5
H-6b	2.93 m	2.80 d (15.4)	C-7	40.9	45.4
H-7	2.83 m	2.68 m	C-8	41.1	45.1
H-8	2.90 m	2.44 d (11.2)	C-9	86.6	90.0
H-9	-	-	C-10	47.1	43.7
H-11a	4.68 dd (2.0, 4.8)	1.84 m	C-11	59.5	22.4
H-11b	-	2.05 ddd (2.0, 4.7, 15.2)	C-12	37.0	26.9
H-12a	1.98 m	1.56 m	C-13	45.0	44.0
H-12b	2.54 m	1.61 m	C-14	41.3	42.7
H-14	2.46 m	1.33 m	C-15	23.6	23.4
H-15a	1.57 m	1.49 m	C-16	35.3	35.2
H-15b	2.32 m	1.84 m	C-17	95.9	94.8
H-16a	1.97 m	1.92 m	C-18	17.2	13.4
H-16b	2.33 m	2.31 m	C-19	26.1	19.1
H-18	1.30 s	0.99 s	C-20	31.5	31.0
H-19	1.86 s	1.40 s	C-21	29.1	29.0
H-20a	2.06 m	1.91 m	C-22	176.3	176.2
H-20b	2.39 m	2.35 ddd (3.6, 5.6, 13.0)	C-23	172.3	176.5
H-21a	2.48 m	2.60 ddd (6.7, 9.2, 17.7)	C-24	52.2	-
H-21b	2.60 m	2.51 m			
H-24	3.61 s	-			

^1^ Values in parentheses give δ, multiplicity and *J* in Hz.

**Table 8:** ^1^H- and ^13^C-NMR spectral data for (7α,11α,17α)-9,11-epoxyester **2**, (7β,11α,17α)-9,11-epoxyester **2a** and (7α,11α,12α,17α)-11,12-epoxyester **2b**.

^1^H-NMR ^1^				^13^C-NMR			
Proton	2	2a	2b	Carbon	2	2a	2b
H-1a	1.42 m	1.46 ddd (2.8, 5.2, 13.2)	2.02 m	C-1	26.7	27.4	34.7
H-1b	2.19 m	2.07 m	2.30 ddd (2.6, 5.0, 13.4)	C-2	33.0	33.2	33.4
H-2a	2.43 m	2.39 m	2.47 dddd (0.8, 2.6, 5.0, 17.5)	C-3	197.9	197.9	198.2
H-2b	2.47 m	2.44 m	2.52 ddd (5.0, 14.3, 17.5)	C-4	127.0	126.8	126.6
H-4	5.89 s	5.83 d (1.8)	5.81 s	C-5	165.0	163.1	165.3
H-6a	2.72 m	2.90 m	2.64 m	C-6	34.8	36.2	35.5
H-6b	2.72 m	2.89 ddd (2.2, 12.7, 15.3)	2.64 m	C-7	41.2	46.3	41.7
H-7	2.89 m	2.67 ddd (3.9, 11.2, 12.6)	2.79 m	C-8	38.6	38.8	35.0
H-8	2.49 dd (4.7, 11.2)	2.59 m	2.00 m	C-9	65.2	67.1	45.9
H-9	-	-	2.04 m	C-10	39.7	40.0	38.2
H-11	3.12 d (5.4)	3.28 d (5.4)	3.02 d (4.0)	C-11	51.4	53.6	50.4
H-12a	1.70 dd (5.4, 14.6)	1.72 dd (5.4, 14.6)	3.05 d (4.0)	C-12	30.9	30.7	56.7
H-12b	1.84 d (14.6)	1.86 d (14.6)	-	C-13	43.8	44.3	47.2
H-14	1.89 m	1.64 m	1.92 m	C-14	37.2	39.4	37.4
H-15a	1.51 m	1.51 m	1.51 m	C-15	22.0	22.4	21.2
H-15b	2.03 m	1.51 m	1.90 m	C-16	34.9	35.1	34.5
H-16a	1.88 m	1.76 m	1.90 m	C-17	94.5	93.9	94.0
H-16b	2.21 m	2.15 m	2.18 m	C-18	16.1	16.5	14.2
H-18	1.02 s	1.02 s	1.09 s	C-19	22.2	21.0	17.9
H-19	1.50 s	1.54 s	1.30 s	C-20	30.8	30.9	31.9
H-20a	1.94 ddd (7.0, 9.6, 13.2)	1.89 m	2.00 m	C-21	28.8	29.0	28.8
H-20b	2.31 ddd (6.5, 9.5, 13.2)	2.28 ddd (6.7, 9.5, 13.2)	2.73 ddd (7.5, 8.9, 12.9)	C-22	176.1	176.1	176.7
H-21a	2.50 ddd (6.9, 9.4, 17.9)	2.50 m	2.60 m	C-23	172.5	174.6	172.5
H-21b	2.60 ddd (6.7, 9.6, 17.9)	2.60 m	2.60 m	C-24	51.5	52.2	51.7
H-24	3.65 s	3.71 s	3.66 s				

^1^ Values in parentheses give δ, multiplicity and *J* in Hz.

**Table 9:** Summary of crystallographic data and structure refinement for the isomeric (7β,11α,17α)-9,11-epoxyester **2a** and (7α,11α,12α,17α)-11,12-epoxyester **2b**.

Identification Code	2a	2b
Chemical formula	C_24_H_30_O_6_	C_24_H_30_O_6_
Molecular weight	414.48 g/mol	414.48 g/mol
Temperature	100(2) K	100(2) K
Wavelength	0.71073 Å	0.71073 Å
Crystal system, space group	Monoclininc, P2_1_	Orthorhomblic, *P*2_1_2_1_2_1_
Unit cell dimensions	a = 6.0294(5) Å	a = 6.377(2) Å
	b = 10.5733(8) Å	b = 15.477(3) Å
	c = 16.3548(12) Å	c = 20.886(4) Å
	α = 90°	α = 90°
	β = 99.604(2)°	β = 90°
	γ = 90°	γ = 90°
Volume	1028.02(14) Å^3^	2061.4(9) Å^3^
*Z*, Calculated density	2, 1.339 mg/m^3^	4, 1.336 mg/m^3^
Absorbtion coefficient	0.095	0.095
*F*(000)	444	888
Crystal size	0.30 × 0.20 × 0.02 mm	0.60 × 0.27 × 0.12 mm
Theta range for data collection	2.30°–27.48°	3.21°–25.23°
Limiting indices	−7 ≤ *h* ≤ 7	−7 ≤ *h* ≤ 7
	−13 ≤ *k* ≤ 13	−18 ≤ *k* ≤ 18
	−21 ≤ *l* ≤ 21	−25 ≤ *l* ≤ 24
Reflections collected/unique	36785/4712	9774/3635
Absorption correction	semi-empirical from equivalents	None
Max. and min. transmission	0.9981 and 0.9720	0.989 and 0.945
Refinement method	full-matrix least-squares on *F*^2^	full-matrix least-squares on all *F*^2^
Data/restraints/parameters	4712/1/274	3635/0/271
Goodness-of-fit on *F^2^*	1.093	1.057
Final *R*1/*wR2* indices [*I* > 2sigma(*I*)]	0.0391/0.1014	0.0371/0.0942
*R1/wR2* indices (all data)	0.0408/0.1024	0.0427/0.0964
Largest diff. peak and hole	0.261 and −0.179 e/Å^3^	0.220 and −0.192 e/Å^3^
